# Hoffmeister Effect Optimized Hydrogel Electrodes with Enhanced Electrical and Mechanical Properties for Nerve Conduction Studies

**DOI:** 10.34133/research.0453

**Published:** 2024-08-14

**Authors:** Yue Zhang, Yijia Hu, Bin Xie, Ganguang Yang, Zhouping Yin, Hao Wu

**Affiliations:** Flexible Electronics Research Center, State Key Laboratory of Digital Manufacturing Equipment and Technology, School of Mechanical Science and Engineering, Huazhong University of Science and Technology, Wuhan 430074, China.

## Abstract

Flexible epidermal electrodes hold substantial promise in realizing human electrophysiological information collections. Conventional electrodes exhibit certain limitations, including the requirement of skin pretreatment, reliance on external object-assisted fixation, and a propensity of dehydration, which severely hinder their applications in medical diagnosis. To tackle those issues, we developed a hydrogel electrode with both transcutaneous stimulation and neural signal acquisition functions. The electrode consists of a composite conductive layer (CCL) and adhesive conductive hydrogel (ACH). The CCL is designed as a laminated structure with high conductivity and charge storage capacity (CSC). Based on the optimization of Hoffmeister effect, the ACH demonstrates excellent electrical (resistivity of 3.56 Ω·m), mechanical (tensile limit of 1,650%), and adhesion properties (peeling energy of 0.28 J). The utilization of ACH as electrode/skin interface can reduce skin contact impedance and noise interference and enhance the CSC and charge injection capacity of electrodes. As a proof of concept, peripheral nerve conduction studies were performed on human volunteers to evaluate the as-fabricated hydrogel electrodes. Compared with the commercial electrodes, our hydrogel electrodes achieved better signal continuity and lower distortion, higher signal-to-noise ratio (~35 dB), and lower stimulation voltages (up to 27% lower), which can improve the safety and comfort of nerve conduction studies.

## Introduction

From the perspective of neuroelectrophysiology, differences in ion concentrations inside and outside the cell membrane and ion transport across the membrane generate electrophysiological signal [1]. Monitoring these electrophysiological signals by electronic devices facilitate the comprehensive understanding of function and status of biological tissues [2], which is important for disease prevention and treatment [3,4]. Among them, nerve conduction studies are used to diagnose and evaluate a wide range of neuromuscular diseases by measuring the electrical activity generated by nerves or muscles when the nerve trunk is stimulated, and they are an important criterion for determining peripheral neuropathy [5]. However, rigid electrodes, which are currently used in clinical medicine, cannot achieve conform contact with skin as their mechanical properties are contradictory to the softness and moistness of human tissues [6].

With the development of wearable technology, it is possible to eliminate bulky and rigid medical devices by using lightweight, flexible electrodes to accomplish nerve conduction studies, enabling bidirectional interaction between biological tissues and external devices [7]. To achieve stable and tight contact with biological tissues, flexible electrodes should preferably have low Young’s modulus [8,9], high stretchability [10,11], strong self-adhesion capability [12–14], and good toughness [15]. In the last few years, innovations in materials and fabrication techniques have triggered the rapid developments in the wearable field [16–19], including the use of materials such as rigid materials [20–22], liquid metal composites [23–26], carbon-based composites [27–30], metal-based nanocomposites [31,32], and conductive polymers [13,33,34], which are used to prepare flexible electrodes through additive fabrication [35–38], chemical deposition [27,39], and screen printing [40,41]. However, for on-skin biosensors, these materials still face drawbacks including low compatibility with skin tissues, weak stretchability, and a lack of interfacial adhesion ability [42]. In tests such as electromyography, in order to realize satisfactory acquisition and stimulation functions, the electrode is required to have characteristics of low interface impedance, stable conformal contact, large charge storage/injection capacity [43], etc. However, most flexible electrodes are only used for the acquisition of signals, while ignoring the importance of nerve stimulation function [44]. In particular, the interface capacitance and charge injection capacity (CIC) of some epidermal electrodes are too low to realize nerve stimulation and signal acquisition simultaneously, which are unpractical for the application in peripheral nerve conduction studies.

**Fig. 1.**
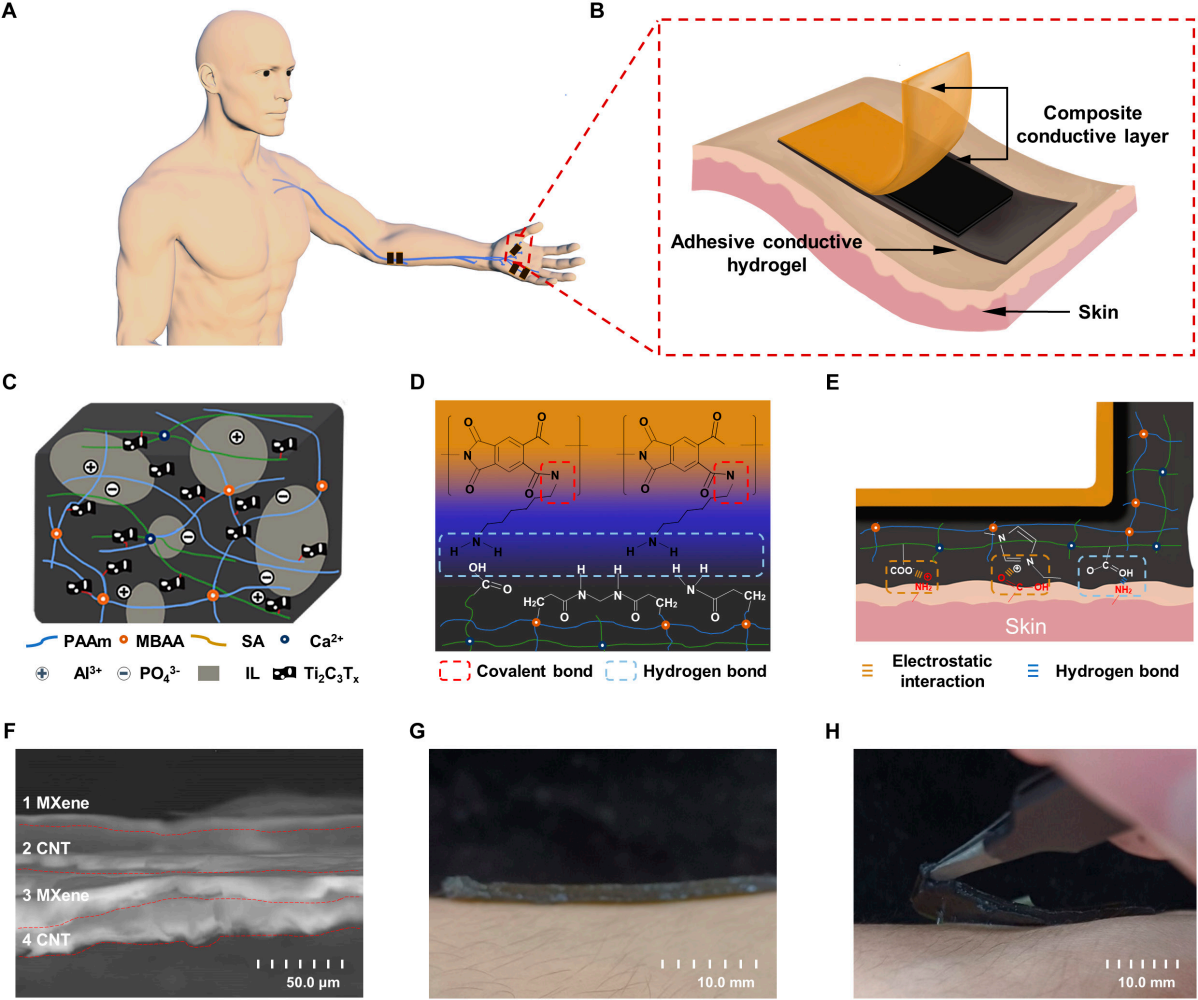
ACH as an interface for on-skin hydrogel electrodes for nerve conduction study. (A) Schematic diagram of the ACH–CCL electrodes for human peripheral nerve conduction study. The electrode combines both transcutaneous stimulation and nerve signal acquisition. (B) Schematic diagram of the ACH–CCL electrode attached to the human skin. (C) Schematic of the composition of ACH with ionic liquid replacement after the introduction of functional materials in the PAAm–SA bipolymer network. (D) Cross-linking mechanism between CCL and ACH. (E) Adhesion mechanism of ACH with human skin. (F) Scanning electron microscopy cross-sectional view of CCL. (G) ACH–CCL electrode adhered onto the surface of human skin. (H) Coupling effect between ACH and human skin when peeling off the hydrogel electrode.

Herein, we developed a hydrogel electrode with both transcutaneous stimulation and nerve signal acquisition functions, with a double-layer structure of adhesive conductive hydrogel (ACH) interface and composite conductive layer (CCL), to realize its application in nerve conduction studies (Fig. 1A). In polyacrylamide (PAAm)/sodium alginate (SA) dual-network hydrogels, we utilized the Hofmeister effect to induce polymer chain entanglement, reduce the volume of the cavity around the polymer backbone, increase the surface tension of the cavity, weaken the bonding between the polymer and water [45], and thus enhance the dispersion of the conductive material (e.g., MXene) in the polymer network [46]. Importantly, the use of ionic liquid (1-hexyl-3-methylimidazolium chloride [HMImCl]) to displace the water in the hydrogel greatly enhances the ionic conductivity of the hydrogel and renders it with low modulus, high stretchability, and high viscosity, which realizes tight adhesion onto human skin. By constructing the laminated structure of MXene/carbon nanotube (CNT), the mechanical properties of CCL are dramatically improved. The laminated structure provides a larger charge attachment space, which enhances the charge storage capacity (CSC) and CIC. Compared with commercial electrodes (CEs), our ACH–CCL electrodes have stronger conformal adhesion and noise suppression properties, with lower contact impedance and higher signal-to-noise ratio (SNR). Meanwhile, the ACH–CCL electrode also shows strong ability in signal stimulation, with higher CSC, stronger charge injection ability, and better stimulation effect. The application of ACH–CCL electrodes in peripheral nerve conduction studies results in lower distortion of the acquired signals and improved stimulation performance, which enhances the safety and comfort of electrical stimulation, demonstrating greater potential for applications in electrophysiological monitoring.

## Results

### Design principle of hydrogel electrodes

As shown in Fig. 1B, the ACH–CCL electrode adopts the form of combining electrode material and hydrogel interface, which consists of CCL and ACH from top to bottom. The CCL consists of a polyimide (PI) substrate layer and a MXene/CNT conductive layer in a laminated structure (Fig. S1). The use of ACH as the electrode interface improves the charge injection effect of the electrodes and the signal fidelity [47]. Based on the enhancement of hydrogel’s electrical and mechanical properties by the Hofmeister effect, we introduced aluminum phosphate (AlPO_4_), Ti_3_C_2_T*_x_*, and HMImCl into the PAAm–SA bipolymer network (Fig. 1C and Fig. S2). As a strong Hofmeister effect ion, PO_4_^3−^ electrolyzed by AlPO_4_ can increase the surface tension of the cavity around the polymer backbone, interfere with the hydrophobic hydration of the macromolecules [48], and induce more Ti_3_C_2_T*_x_* lamellae to be dispersed and connected to the polymer network through hydrogen bonding interactions, enhancing the CSC and mechanical properties of the hydrogel. PO_4_^3−^ is also able to induce entanglement of polymers chains [49], reducing the cavity voids of the hydrogel network [50] and lowering the swelling rate. By using HMImCl to replace water in the hydrogel, the adhesion and dehydration resistance of the hydrogel can be enhanced while Ti_3_C_2_T*_x_* is less dispersed with water.

Figure 1D outlines the mechanism by which CCL and ACH form tight cross-links through 1,6-diaminohexane (DAH), which is sufficient to support the stable use of hydrogel electrodes (Fig. S3). Among them, the CCL is based on PI, which contains an imide ring on its main chain and can react with DAH in an amine-functionalized imide ring. In ACH, SA containing carboxyl groups, acrylamide (AAm) containing amino groups, and N,N′-methylenebisacrylamide (MBAA) containing imino groups in the hydrogel can form hydrogen bonds with the amino group on DAH. Figure 1E illustrates the adhesion mechanism of the hydrogel layer to the skin, which mainly consists of electrostatic interaction and hydrogen bond. Figure 1F shows a scanning electron microscopy cross-sectional image of the CCL, showing the laminated structure of Ti_3_C_2_T*_x_* and CNT, which enables the hydrogel electrodes to maintain the conductive stability under bending deformation and have higher CSC and CIC. The ACH–CCL electrodes have good adhesion ability and can be tightly adhered to the surface of human skin without utilizing external assistance (Fig. 1G). The robust adhesion of the hydrogel–skin interface can be observed when it is peeled from a subject’s arm (Fig. 1H).

### Physical properties

**Fig. 2.**
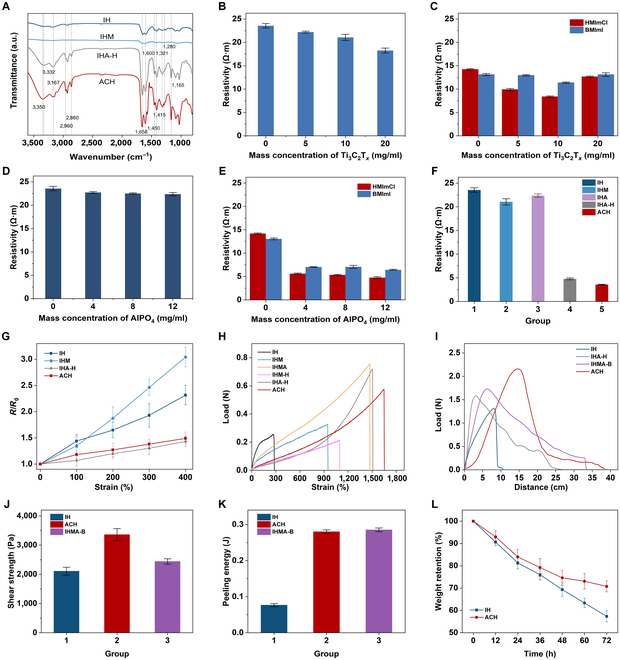
Physical properties characterizations of ACH. (A) FTIR-ATR spectra of ACH, IHA-H, IHM, and IH. (B) Variation of IHM resistivity with the mass fraction of MXene. (C) Variation of IHM resistivity with ionic liquid treatment. (D) Variation of IHA resistivity with the mass fraction of AlPO_4_. (E) Changes in IHA resistivity after treatment with ionic liquid. (F) Resistivity comparison of IH, IHM, IHA, IHA-H, and ACH. (G) Resistance change rate of IH, IHM, IHA-H, and ACH when stretched. (H) Tensile load–strain curves of IH, IHM, IHMA, IHM-H, IHA-H, and ACH. (I) Tensile load–distance curves of IH, IHMA-B, and ACH. (J) Maximum shear strength of IH, ACH, and IHMA-B. (K) Stripping energy of IH, ACH, and IHMA-B. (L) Dewatering resistance test of IH and ACH.

Here, the hydrogel containing only the PAAm–SA dual network is referred to as IH. On the basis of IH, hydrogels with only MXene are called IHM, hydrogels with only AlPO_4_ are called IHA, and the hydrogels with both MXene and AlPO_4_ are called IHMA. The addition of “-H” or “-B” after the name denotes the presence of HMImCl or 1-butyl-3-methylimidazolium iodide (BMImI), respectively. Hydrogels containing both MXene, AlPO_4,_ and HMImCl are referred to as ACH. With Fourier transform infrared spectroscopy-attenuated total reflectance (FTIR-ATR) spectroscopy and x-ray photoelectron spectroscopy (XPS), we characterized ACH, IHA-H, IHM, and IH (Fig. 2A; XPS is shown in Fig. S4 in supporting information). A detailed discussion in Note S1 verifies the presence of these functional components in ACH. It can be hypothesized that there is a strong interaction between Ti_3_C_2_T*_x_* and the hydrogel network, and a large number of hydroxyl groups on its surface form hydrogen bonds with the amino groups supplied by HMImCl, which enhances the mechanical properties of ACH. The large absorption peak at 1,658 cm^−1^ is the stretching vibrational band of the carbonyl group, which is absent in IH and IHM due to the absence of HMImCl, suggesting that after solvent replacement, the ionic liquid enters the hydrogel system at a fairly high level. To further quantitatively evaluate the effects of functional materials on the electrical properties of ACH, we prepared the corresponding hydrogels by adjusting the mass fraction of different functional materials in IH. Figure 2B illustrates that the resistivity of the IHM shows a trend of decreasing from 22.55 to 18.24 Ω·m as the mass fraction of Ti_3_C_2_T*_x_* increases. The conductivity of IHM treated with HMImCl and BMImI, respectively, shows a decreasing and then increasing trend, and the resistivity is lowest at a Ti_3_C_2_T*_x_* mass fraction of 10 mg/ml (Fig. 2C). This is because that the effect of Ti_3_C_2_T*_x_* in enhancing the electrical conductivity of IH is weaker than that of ionic liquids, and when the mass fraction of Ti_3_C_2_T*_x_* is low, it is able to utilize the hydrogen bonding to enhance the displacement effect of ionic liquids, whereas with the increase of the mass fraction of Ti_3_C_2_T*_x_*, it excessively occupies the position of ionic liquids in the cavities of the hydrogel network, which results in the diminution of the electrical conductivity of hydrogel. Figure 2D shows that the resistivity of IHA decreases from 23.55 to 22.37 Ω·m with increasing AlPO_4_ mass fraction. However, the resistivity of IHA shows a marked decrease after ionic liquid replacement, and the resistivity of IHA treated with HMImCl decreased from 14.2 to 4.77 Ω·m, which was better than that of BMImI. After treatment with ionic liquids, the resistivity of IHA decreases more than that of IH because PO_4_^3−^ induces a strong Hofmeister effect, which reduces the swelling rate of the hydrogel (Fig. S5) and enhances the displacement of the ionic liquid (Fig. 2E). Comparison of the resistivities of IH, IHM, IHA, IHA-H, and ACH (Fig. 2F) indicates that the addition of PO_4_^3−^ and treatment with HMImCl could enhance the ionic liquid solvent displacement using the Hofmeister effect, while the addition of an appropriate amount of Ti_3_C_2_T*_x_* could reduce the resistivity of the hydrogel to an even greater extent (3.56 Ω·m). Finally, the rate of resistance change during stretching of IH, IHM, IHA-H, and ACH was characterized to investigate the effect of functional materials on the mechanical properties of hydrogels (Fig. 2G). IHM has a higher rate of resistance change during stretching because Ti_3_C_2_T*_x_* is connected to the hydrogel network through hydrogen bonding, which will be displaced with the hydrogel deformation. The lower rate of resistance change of IHA-H is partly due to the fact that the Hoffmeister effect tightens up the hydrogel network and partly due to the fact that the electrical conductivity of ionic liquids has a smaller change with deformation.

The rate of change of resistance of IHA-H is only slightly smaller than that of ACH, but the resistivity is noticeably higher than that of ACH (~25%), and taken together, the overall electrical performance of ACH is better. In summary, we chose 10 mg/ml Ti_3_C_2_T*_x_* and 12 mg/ml AlPO_4_ as the conductive functional materials to prepare hydrogels and performed solvent substitution with HMImCl to obtain ACH, thus optimizing the conductivity of hydrogels.

To investigate the effect of functional materials on mechanical properties, we selected IH, IHM, IHMA, IHM-H, IHA-H, and ACH for tensile comparison experiments. As shown in Fig. 2H, the modulus and strain-at-break of IHMA are larger than that of IHM, which indicates that the mechanical properties of the hydrogel subjected to the Hofmeister effect are enhanced. After the addition of ionic liquid, ACH exhibited the maximum tensile limit of 1,650%, while the maximum load was lower than that of IHA-H, which may be due to the multiple anchoring interactions (hydrogen bonding, electrostatic coupling, etc.) of MXene with the polymer long chains and high-valence ions, which weaken the strong electrostatic interactions of the high-valence ions (generated by AlPO_4_) and promote the energy dissipation of the polymer network [51], resulting in a lower network fracture strength and weaker loading capacity. This confirms that hydrogel schemes optimized for electrical properties can also contribute to the enhancement of mechanical properties.

In addition, ionic liquids play a major role in influencing the adhesion properties of hydrogels, and we prepared the samples accordingly by adjusting the type and presence of ionic liquids. As shown in Fig. 2I, the tensile load-distance curves of the hydrogels were obtained by the standard shear test method (ASTM F22505), the test substrate was freshly dehairing pig skin (Fig. S6), and the maximum shear strength of the hydrogels can be calculated by Eq. 1:

$$\sigma_{\text{shear}}=\frac{F_{\max}}{w\cdot l}$$ (1)

Figure 2J demonstrates that the shear strength of ACH is the greatest, with an enhancement of about 59.5% (up to 3,360 ± 205 Pa) over the IH, whereas the BMImI-treated hydrogel (IHMA-B) has an enhancement of only 15.8% (up to 2,440 ± 93 Pa). The peeling energy of the hydrogels can be calculated by Eq. 2:

$$W_{\text{strip}}=\int F\cdot \Delta x$$ (2)

The peeling energy of ACH is about 265.4% higher (0.28 J) than that of IH (Fig. 2K). Comparative experiments show that the hydrogels treated with ionic liquids have better adhesion properties. This is due to the ability of ionic liquids to bind to carboxyl groups on the skin through electrostatic interaction, achieving adhesion enhancement. Figure 2L shows the changes in the relative mass of ACH and IH in the experimental environment at 15°C and 40% relative humidity, and it can be found that ACH is more resistant to dehydration. After 72 h, the relative mass of IH decreases to 57.3%, whereas the relative mass of ACH is 70.8%. In addition, there is no residue or irritation on human skin even after 20 peeling cycles or 1 h of ACH attachment, demonstrating the great skin friendliness of ACH (Fig. S7).

### Hydrogel electrodes with ACH interface and CCL

In neurodiagnostics, on-skin electrodes with both collecting and stimulating capabilities are in urgent demand. The ACH is applied as the functional interface of the on-skin hydrogel electrodes, while CCL is employed as the conductor of the electrodes, which is able to enhance the stimulation performance of the electrodes. The 2D conductive material MXene, especially Ti_3_C_2_T*_x_*, has high conductivity, large CIC, and rich surface functional groups, which is convenient for integration with other materials. At the same time, the use of CNT with high CSC as an additive material is combined with Ti_3_C_2_T*_x_*, so that the conductive layer has good electrical conductivity and mechanical properties. The laminated structure design can comprehensively improve the electrical properties and bending resistance of the conductive layer.

**Fig. 3.**
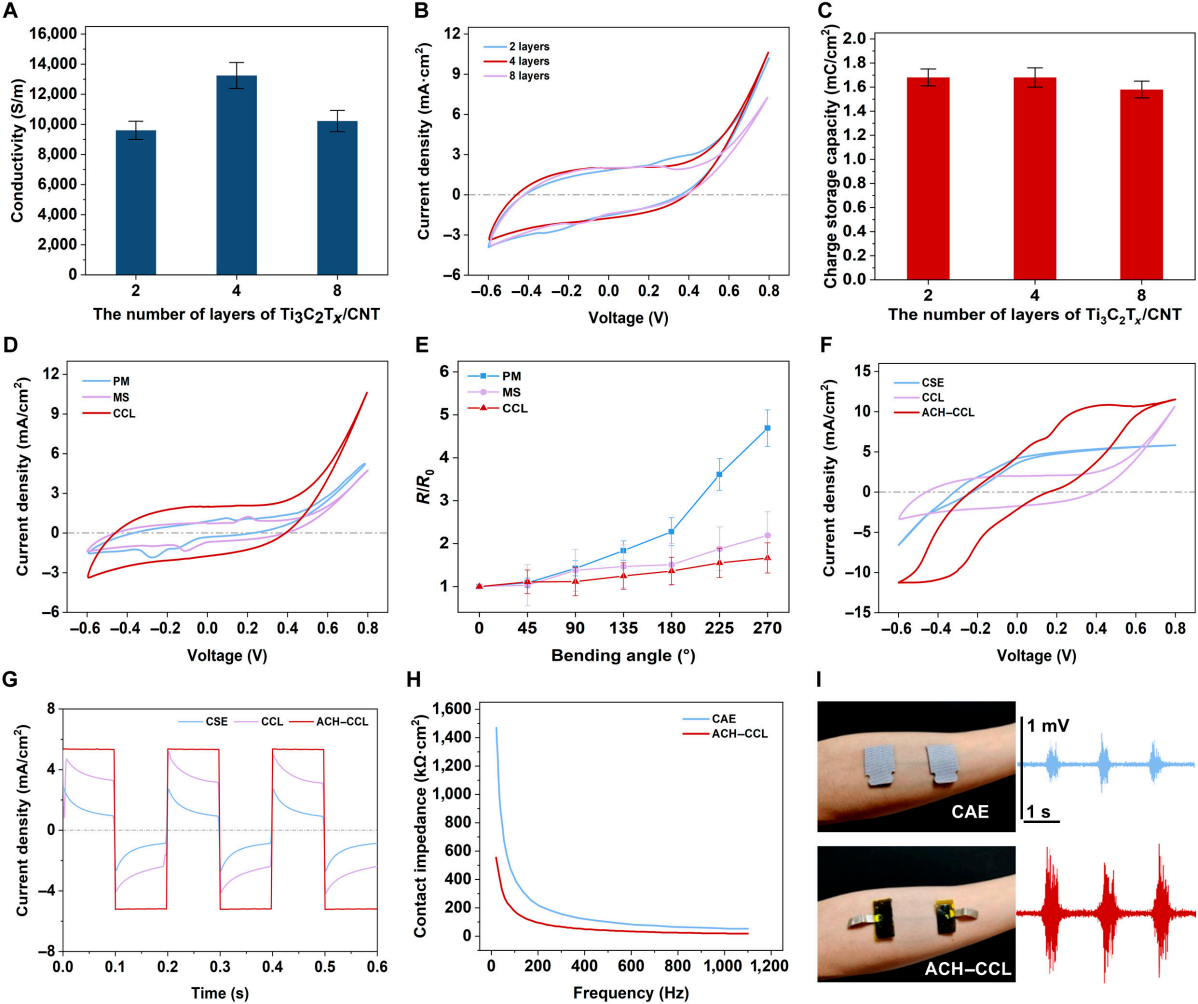
Performance characterizations of hydrogel electrodes with ACH interface and CCL. (A) Comparison of the conductivity of CCL with different laminating layers. (B) Voltage–current density curves of CCL with different layers. (C) Comparison of CSC of CCL with different layers. (D) Voltage–current density curves of PM, MS, and CCL. (E) Rate of resistance change when PM, MS, and CCL were bent. (F) Voltage–current density curves of CSE, CCL electrode, and ACH–CCL electrode. (G) Time–charge density curves of CSE, CCL electrode, and ACH–CCL electrode. (H) Comparison of contact impedance between CAE and ACH–CCL electrode. (I) CAEs and ACH–CCL electrodes were used to collect electromyogram signals.

To investigate the effect of the number of MXene/CNT layers on the electrical properties, we conducted comparative experiments with different numbers of conductive layers. With the area, thickness, and total amount of CNT and Ti_3_C_2_T*_x_* same for each set of conductive layers, by only changing the number of layers of the laminated structure to 2, 4, and 8, the conductivity of the conductive layers is shown in Fig. 3A. It can be noticed that the conductivity of the CCL shows a fluctuation of increasing and then decreasing with the increase of the number of layers. The voltage–current density variation curve of the CCL is shown in Fig. 3B, and the CSC is obtained by calculating the integral of the current over time (Fig. 3C). It can be found that the number of layers has little effect on the CSC of the conductive layers but shows a trend of slightly decreasing charge capacity with increasing number of layers. Although the number of layers of the laminated structure is different, the total amount of CNT and Ti_3_C_2_T*_x_* is identical, and the difference in the conductive properties may be attributed to the effect of the air gaps between the CNT and Ti_3_C_2_T*_x_* layers, and either too large or too small interlayer gaps may affect the electron mobility efficiency and lead to the performance changes. When the number of Ti_3_C_2_T*_x_*/CNT conducting layers is 4, the CCL has the highest conductivity and higher CSC, indicating an optimal overall performances. To further demonstrate the enhancement of the electrical properties of the CCL by the laminated structure, we compared the electrical properties of the pure Ti_3_C_2_T*_x_* conductive layer (PM), the Ti_3_C_2_T*_x_* and CNT mixed-structure conductive layer (MS), and the CCL, while maintaining equal total mass or ratio. Both PM and MS have much lower CSC than CCL, suggesting that the laminated structure creates a larger charge storage space (Fig. 3D). Consistent with expectations, the electrical conductivity of CCL is lower than that of PM but higher than that of MS (Fig. S8). The incorporation of CNT or the design of the laminated structure are both able to reduce the resistivity change rate when the conductive layer was bent, which results in the strongest bending resistance of CCL (Fig. 3E). By combining those effects together, the CCL has the best electrical performance.

CEs including commercial stimulation electrodes (CSEs) and commercial acquisition electrodes (CAEs) were used as comparison with the ACH–CCL electrodes. As shown in Fig. 3F, the voltage–current density variation curves of the CSE, CCL electrode, and ACH–CCL electrode were measured, and the CSC of each electrode was calculated to be 0.43, 1.92, and 3.325 mC/cm^2^, respectively. This is due to the fact that the conductive functional materials in the ACH provide more space for the charge to attach. The time–charge density curves of the 3 electrodes were measured (Fig. 3G), and the CIC of each electrode was calculated to be 0.134, 0.358, and 0.522 mC/cm^2^, respectively, which indicates that the ACH–CCL electrodes have enhanced CIC. At the same time, the ACH–CCL electrode has a flatter CIC curve, meaning a milder stimulation effect. In addition, due to the good compliance of the ACH interface, the contact impedance of the ACH–CCL electrode (22.82 kΩ·cm^2^, 1,080 Hz) is lower than that of the CAE (52.43 kΩ·cm^2^, 1,080 Hz), which also contributes to the improvement of the SNR of the acquired signal (Fig. 3H). Both CAE and ACH–CCL electrodes were used to acquire EMG signals from the lateral muscle groups of the human forearm (Fig. 3I). It is evident that the ACH–CCL electrodes acquired better signal quality, which is attributed to the ability of ACH to act as an ion channel to enhance charge transfer. Meanwhile, the ACH interfacial layer has stronger conformal adhesion and better noise suppression ability under wet adhesion.

### Peripheral nerve conduction studies

Peripheral nerve conduction study, including examination of motor and sensory nerve conduction, can assist the clinician in the diagnosis of neurologic disorders. In this work, we conducted the medical examination of carpal tunnel syndrome and elbow tunnel syndrome based on the as-fabricated ACH–CCL electrodes.

**Fig. 4.**
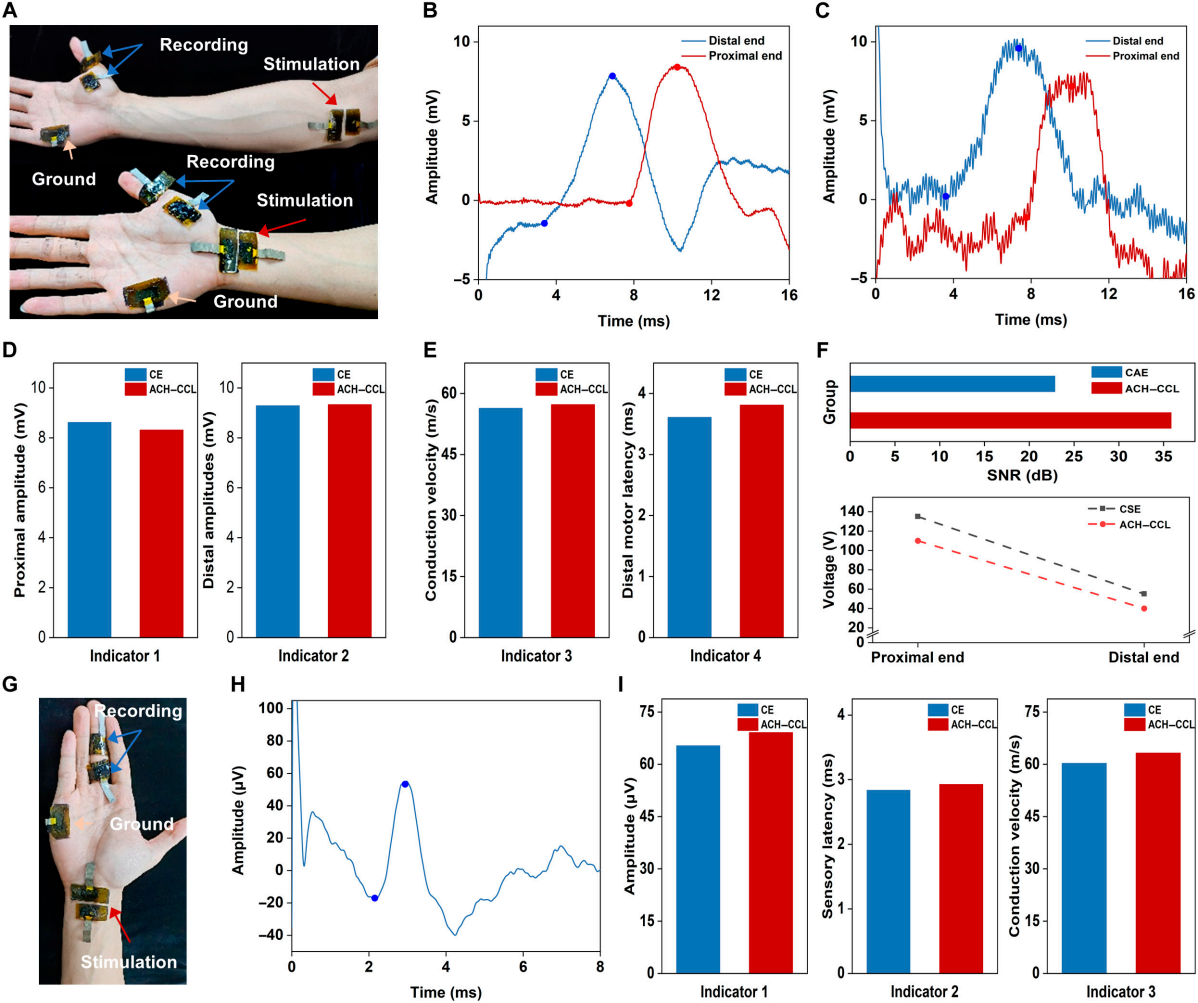
Tests for carpal tunnel syndrome. (A) For the median nerve motor branch test, the ACH–CCL electrodes were used as the acquisition electrodes, stimulation electrodes, and ground electrode at the same time, and distal stimulation and proximal stimulation of the motor nerve were achieved. (B and C) Waveform images of the motor branch of the median nerve were acquired by the ACH–CCL electrodes and the CE, respectively. (D and E) Comparison of 4 test parameters (proximal amplitude, distal amplitude, conduction velocity, and distal motor latency) acquired by the CE and the ACH–CCL electrodes. (F) Comparison of SNR and voltages required for proximal/distal stimulation of CE (CAE/CSE) and ACH–CCL electrodes. (G) For testing of the sensory branch of the median nerve, the ACH–CCL electrodes were used as the acquisition electrodes, stimulation electrodes, and ground electrode simultaneously. (H) ACH–CCL electrodes acquired waveform images of the median nerve sensory branch. (I) Comparison of voltage amplitude, sensory latency, and conduction velocity acquired by CE and ACH–CCL electrodes.

Carpal tunnel syndrome is one of the most common peripheral nerve entrapment disorders. The motor branch of the median nerve was examined using ACH–CCL electrodes and CEs, respectively, which entailed placing the acquisition electrodes in the center of the thumb abductor digitorum brevis muscle belly and distal to the thumb, the proximal stimulating electrodes just above the brachial artery at the elbow, and the distal stimulating electrodes at the wrist with the carpal tunnel off the radial side of the carpal tunnel (Fig. 4A), and the data from the electromyograph were used for comparison. The waveforms of the motor branch of the median nerve (distal stimulation and proximal stimulation) acquired by the ACH–CCL electrodes are shown in Fig. 4B, and the waveforms acquired by the CAE are shown in Fig. 4C. ACH–CCL electrodes capture smoother signals with lower noise. Comparing the electromyograph results (Table S1), the 4 tested parameters (distal amplitude, proximal amplitude, conduction velocity, and distal motor latency) of the motor branch of the median nerve (Fig. 4D and E) indicate that the subjects were within the normal range. Ensuring that all other conditions were the same, the SNR value of the acquired signal from the ACH–CCL electrodes (35.85 dB) is much higher than that of the CAE (22.86 dB). To achieve the same hyperstimulation effect, we applied to the ACH–CCL electrodes a voltage (110 V proximal, 40 V distal) lower than that of the CSE (135 V proximal, 55 V distal), which provides a prominent enhancement in both safety and comfort for the subjects (Fig. 4F). For the examination of the sensory branch of the median nerve, the acquisition electrodes were placed at the middle finger joint, and the stimulation electrodes were placed at the distal end of the motor branch (Fig. 4G); the waveforms acquired by the ACH–CCL electrodes are shown in Fig. 4H. Comparing the waveforms acquired by the ACH–CCL electrodes with those acquired by the CAE (Fig. S9), the waveforms acquired by the ACH–CCL electrodes are smoother under the same processing, indicating that our electrodes have better acquisition capability and lower noise, which is attributed to fact that the CCL has good bending stability, while the ACH has excellent adhesion and low modulus, and the use of the ACH as the electrode–skin interface enables stable conformal contact with the skin, decreases the contact impedance, and reduces the interference of the skin micromotion on signal acquisition [52]. At the same time, a large number of ions (generated by AlPO_4_ and HMImCl) and conductive substances (MXene) act as intermediate channels to achieve capacitive coupling between the CCL and the skin, which further reduces the contact impedance and attenuates the noise generation [47,53]. Parameters such as amplitude, sensory latency, and conduction velocity acquired by both electrodes (Fig. 4I) are consistent with those obtained by the electromyograph (Table S2).

**Fig. 5.**
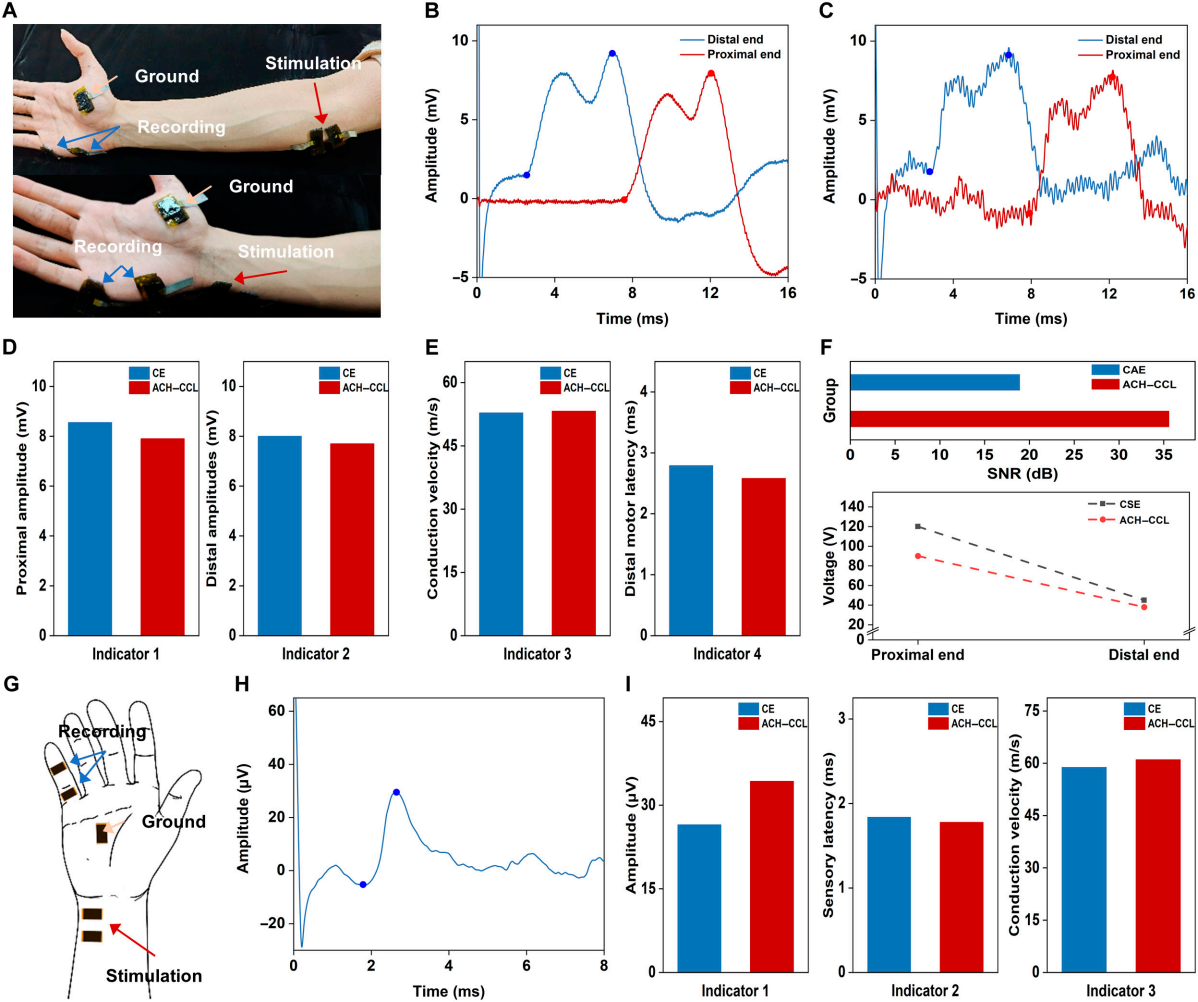
Tests for cubital tunnel syndrome. (A) Photograph of the ulnar nerve motor branch test, in which the ACH–CCL electrodes were used as the acquisition electrodes, stimulation electrodes, and ground electrode at the same time, and distal stimulation and proximal stimulation of the motor nerve were achieved. (B and C) Waveform images of the motor branch of the ulnar nerve were acquired by the ACH–CCL electrodes and the CE, respectively. (D and E) Proximal amplitude, distal amplitude, conduction velocity, and distal motor latency acquired by the CE and the ACH- CCL electrodes. (F) SNR and voltages required for proximal/distal stimulation of CE (CAE/CSE) and ACH–CCL electrodes. (G) Testing of the sensory branch of the ulnar nerve. The ACH–CCL electrodes were used as the acquisition electrodes, stimulation electrodes, and ground electrode simultaneously. (H) ACH-CCL electrodes acquired waveform images of the ulnar nerve sensory branch. (I) Voltage amplitude, sensory latency, and conduction velocity acquired by CE and ACH–CCL electrodes.

Cubital tunnel syndrome is the second most prevalent clinical condition after carpal tunnel syndrome, which is caused by progressive damage to the ulnar nerve due to compression at the elbow. In the examination of the ulnar nerve, the signal acquisition point of the motor branch is on the spreading muscles of the little finger; the proximal stimulation point is below the elbow, along the ulnar nerve trunk and about 5 cm above the medial-superior humeral ankle; and the distal stimulation location is on the ulnar side of the wrist, close to the parallel cross-section of the wrist (Fig. 5A). Again, using the data measured by the electromyograph for comparison, we completed the examination of the ulnar nerve with the ACH–CCL electrodes and CE. The waveforms of the motor branch of the median nerve (distal stimulation and proximal stimulation) acquired by the ACH–CCL electrodes are shown in Fig. 5B, and the waveforms acquired by the CAE are shown in Fig. 5C. The results demonstrate that the ACH–CCL electrodes are more capable of acquiring high-quality signals. Comparing the results measured by the electromyograph (Table S3), the results of the 4 tested parameters (distal amplitude, proximal amplitude, conduction velocity, and distal latency) (Fig. 5D and E) of the subjects showed that all the indexes of the subjects were within the normal range. With other conditions being equal, the SNR of the acquired signals from the ACH–CCL electrodes (35.58 dB) was much higher than that of the CAE (18.92 dB). To achieve the same stimulation effect, we applied to the ACH–CCL electrodes a voltage (90 V proximal, 38 V distal) lower than that of the CSE (120 V proximal, 45 V distal), which proves the improved safety and comfort of the ACH–CCL electrodes (Fig. 5F). For the examination of the sensory branch of the ulnar nerve, the stimulation location of the sensory branch was the same as the distal stimulation location of the motor branch, and the acquisition electrode was located at the little finger joint (Fig. 5G); the waveforms acquired by the ACH–CCL electrodes are shown in Fig. 5H. The amplitude, sensory latency, and conduction velocity results (Fig. 5I) are all within 10% of the electromyograph results (Table S4). From the comparison of the waveforms acquired by the ACH–CCL electrodes with those acquired by the CAE (Fig. S10), it is demonstrated that the ACH–CCL electrodes have greater acquisition capability.

## Discussion

We have developed hydrogel electrodes with a novel ACH with high electrical, mechanical, and adhesion properties at the same time, which can be used as an electrode/skin interface to realize high-quality nerve signal acquisition and stimulation functions to complete the peripheral nerve conduction study in human body. Based on the strong Hofmeister effect induced by PO_4_^3−^, the addition of MXene, and the use of ionic liquids to complete the solvent substitution, the ACH demonstrates both superior electrical (resistivity of 3.56 Ω·m) and mechanical (tensile limit of 1,650%) properties, with an increase in the maximum shear strength of 59.5% (3,360 ± 205 Pa) and an increase in the peel energy of 265.4% (0.28 J). Importantly, ACH has a larger charge attachment space, which not only greatly enhances the charge injection capability but also suppresses noise generation.

By employing ACH as the interfaces of the electrodes, we combined ACH with CCL to prepare ACH–CCL electrodes. Owing to the flexibility and adhesion of the ACH, the contact impedance between ACH–CCL electrodes and skin is reduced. We further applied the ACH–CCL electrodes in the peripheral nerve conduction study of human body. We selected the median and ulnar nerves, which are commonly used for diagnosing specific diseases, and examined their motor and sensory branches, respectively. The results show that compared with the CEs, the ACH–CCL electrodes improved the quality of the nerve signal acquisition while guaranteeing the correct results, and the SNR was increased by at least 40%. In the examination of motor branch, the ACH–CCL electrodes were able to acquire clearer and more accurate waveforms without filtering. For the sensory branch, the ACH–CCL electrodes acquired waveforms with lower distortion. At the same time, ACH–CCL electrodes have stronger stimulation performance, so that the voltage required for the nerve to reach the same level of excitation is cut down, which improves the safety and comfort of the examination and shows great prospect for applications in the field of neurodiagnostics.

## Materials and Methods

### Materials

CNTs were purchased from Xianfeng Nanomaterials Technology Co., Ltd. (Jiangsu, China). Ti_3_C_2_T*_x_* was purchased from Xinen Technology Co., Ltd. (Foshan, China). Polyvinyl alcohol, MBAA, SA, tetramethylethylenediamine, and calcium sulfate (CaSO_4_) were purchased from Aladdin Biochemistry Technology Co. (Shanghai, China). PI was purchased from Bomi Technology Co. AAm was purchased from McLean Biochemistry Technology Co. (Beijing, China). AlPO_4_ was purchased from Alfa Aesar Chemical Co. (Shanghai, China). Ammonium persulfate was purchased from Sinopharm Chemical Reagent Co. (Shanghai, China). HMImCl was purchased from Biotech Pharmaceutical Technology Co. (Shanghai, China).

### Fabrication of the CCL

The CNT dispersion (0.15 wt%, 0.2 ml) was uniformly dropped on the solvent microporous filtration membrane (50 mm in diameter, 0.2 μm in diameter) to cure it into a film by negative pressure extraction filtration. Then, Ti_3_C_2_T*_x_* dispersion solution (5 mg/ml, 0.2 ml) was uniformly dropped on the CNT film, and after curing, a layered conductive layer was obtained. The number of conductive layers could be adjusted by repeating the above steps. Then, the conductive layer was heated in an oven at a constant temperature (70°C, 5 min). Polyvinyl alcohol (10 wt%, 1 ml) solution was spin-coated on the glass plate to form a sacrifice layer, and the prepared conductive layer was placed on the sacrifice layer. Glass rods were dipped in PI solution (20 wt%, 2 ml) and spread over the surface of the conductive layer, then they were placed in a spin-coating machine for spin coating (350 r/min, 90 s). Next, the conductive layer was vacuumed in the vacuum chamber (10 min) to remove the bubbles in the PI solution and then cured in the oven (200°C, 1.5 h). Next, the sample was placed in a water bath wrap at 80°C for 1 h to remove the sacrificial layer. Finally, the conductive layer was immersed in 1,6-hexanediamine solution (10 wt%, 5 ml) for 4 to 6 h and poured into an acrylic mold to finish the preparation of CCL.

### Fabrication of the ACH

The monomer prepolymerization solution was prepared by adding 2 g of AAm, 1.1 mg of MBAA, and 0.2 g of SA to 15 ml of deionized water and stirred with a magnetic stirrer at 400 r/min for 5 to 6 h. Afterwards, 6.5 mg of tetramethylethylenediamine, 180 mg of AlPO_4_, and 150 mg of Ti_3_C_2_T*_x_* were added, shaken for 5 min, and degassed after ultrasonication for 10 min. The cross-linking promotion solution was prepared by adding 650 mg of ammonium persulfate and 112 mg of CaSO_4_ to 10 g of deionized water. Subsequently, 5 ml of monomer prepolymerization solution and 200 μl of cross-linking promotion solution were withdrawn with 2 syringes, connected through luer fittings, mixed homogeneously, and poured into acrylic molds, and reacted for 4 h. After that, the hydrogel was removed from the mold and covered with HMImCl (30 wt%) for solvent replacement for 1.5h to complete the ACH preparation.

### Fabrication of the ACH–CCL electrodes

Preliminary steps are the same as above, using 2 syringes to extract 5 ml of monomer pre-polymerization solution and 200 μl of cross-linking promoter solution, respectively, connected through the luer connector, mixed homogeneously, and then poured into the acrylic mold with CCL, heated at 60°C for 10 to 15 min. After heating, the 2 parts formed a tight whole, which was separated from the mold, and the solvent replacement was done by covering the hydrogel with HMImCl.

### Characterization

Interfacial images of the MXene/CNT laminated structures were observed using a scanning tunneling electron microscope (MIRA4, TESCAN, Czech Republic).

The square resistance and conductivity of CCL were measured using a 4-probe tester (RTS-8, Tianjin Norexinda, China), and the CIC and CSC of ACH–CCL electrodes and CCL were tested using an electrochemical workstation (Autolab PGSTAT302N, Metrohm, Switzerland).

XPS spectra were recorded by an x-ray photoelectron spectrometer (AXIS-ULTRA DLD-600W, Shimadzu-Kratos, Japan).

Fourier transform infrared spectrograms were recorded by a controlled atmosphere Fourier transform infrared spectrometer (Nicolet iS50, Thermo Fisher, USA).

CEs included CSEs (Dantec Keypoint G4, Denmark) and CAEs (LT-8, Shanghai LITU Medical Appliances Co.).

The contact impedance of the CAEs and ACH–CCL electrodes was measured using a LCR meter (E4980AL, Keysight, USA).

In the testing of the motor branch of the median nerve, a USB-6218 acquisition card was used in conjunction with a SC-2002 low-frequency pulser (Shanghai Xinshang Medical Devices, Shanghai, China) in lieu of an electromyograph, with a stimulation frequency of 2 Hz, a pulse width of 0.2 ms, and an acquisition frequency of 30 kHz.

After an explanation of the nature and possible consequences of the studies, all subjects gave fully voluntary and informed consent to participate in the experiment.

## Data Availability

The data that support the plots within this paper and other findings of this study are available from the corresponding authors upon reasonable request.

## Supplementary Materials

Supplementary 1 Figs. S1 to S10 Tables S1 to S4 Note S1

## References

[1] Zhang B, Xie R, Jiang J, Hao S, Fang B, Zhang J, Bai H, Peng B, Li L, Liu Z, et al. Implantable neural electrodes: From preparation optimization to application. J Mater Chem C. 2023;11(20):6550–6572.

[2] Boehler C, Carli S, Fadiga L, Stieglitz T, Asplund M. Tutorial: Guidelines for standardized performance tests for electrodes intended for neural interfaces and bioelectronics. Nat Protoc. 2020;15(11):3557–3578. 33077918 10.1038/s41596-020-0389-2

[3] Choi JS, Lee HJ, Rajaraman S, Kim D-H. Recent advances in three-dimensional microelectrode array technologies for in vitro and in vivo cardiac and neuronal interfaces. Biosens Bioelectron. 2021;171:112687. 33059168 10.1016/j.bios.2020.112687 PMC7665982

[4] Zhu M, Wang H, Li S, Liang X, Zhang M, Dai X, Zhang Y. Flexible electrodes for in vivo and in vitro electrophysiological signal recording. Adv Healthc Mater. 2021;10(17):2100646. 10.1002/adhm.202100646 34050635

[5] Jones LK Jr. Nerve conduction studies: Basic concepts and patterns of abnormalities. Neurol Clin. 2012;30(2):405–427. 22361368 10.1016/j.ncl.2011.12.002

[6] Yuk H, Wu J, Zhao X. Hydrogel interfaces for merging humans and machines. Nature Reviews Materials. 2022;7(12):935–952.

[7] Frank JA, Antonini M-J, Anikeeva P. Next-generation interfaces for studying neural function. Nat Biotechnol. 2019;37(9):1013–1023. 31406326 10.1038/s41587-019-0198-8 PMC7243676

[8] Khan Y, Garg M, Gui Q, Schadt M, Gaikwad A, Han D, Yamamoto NAD, Hart P, Welte R, Wilson W, et al. Flexible hybrid electronics: Direct interfacing of soft and hard electronics for wearable health monitoring. Adv Funct Mater. 2016;26(47):8764–8775.

[9] Xu C, Yang Y, Gao W. Skin-interfaced sensors in digital medicine: From materials to applications. Matter. 2020;2(6):1414–1445. 32510052 10.1016/j.matt.2020.03.020 PMC7274218

[10] Guo R, Bao Y, Zheng X, Zhang W, Liu C, Chen J, Xu J, Wang L, Ma J. Hofmeister effect assisted dual-dynamic-bond cross-linked organohydrogels with enhanced ionic conductivity and balanced mechanical properties for flexible sensors. Adv Funct Mater. 2023;33(12):2213283.

[11] Bai Z, Wang X, Zheng M, Yue O, Huang M, Zou X, Cui B, Xie L, Dong S, Shang J, et al. Mechanically robust and transparent organohydrogel-based e-skin nanoengineered from natural skin. Adv Funct Mater. 2023;33(15):2212856.

[12] Yang J, Bai R, Suo Z. Topological adhesion of wet materials. Adv Mater. 2018;30(25):1800671. 10.1002/adma.201800671 29726051

[13] Tan P, Wang H, Xiao F, Lu X, Shang W, Deng X, Song H, Xu Z, Cao J, Gan T, et al. Solution-processable, soft, self-adhesive, and conductive polymer composites for soft electronics. Nat Commun. 2022;13(1):358. 35042877 10.1038/s41467-022-28027-y PMC8766561

[14] Wu H, Li Z, Xu Z, Huang X, Guo W, Zhao J, Zhang J, Liu S, Tang M, Qiu Y, et al. On-skin biosensors for noninvasive monitoring of postoperative free flaps and replanted digits. Sci Transl Med. 2023;15(693):eabq1634. 37099631 10.1126/scitranslmed.abq1634

[15] Li F, Zhang G, Wang Z, Jiang H, Yan S, Zhang L, Li H. Strong wet adhesion of tough transparent nanocomposite hydrogels for fast tunable focus lenses. ACS Appl Mater Interfaces. 2019;11(16):15071–15078. 30938504 10.1021/acsami.9b02556

[16] Shang K, Gao J, Yin X, Ding Y, Wen Z. An overview of flexible electrode materials/substrates for flexible electrochemical energy storage/conversion devices. Eur J Inorg Chem. 2021;2021(7):606–619.

[17] Huang X, Guo W, Liu S, Li Y, Qiu Y, Fang H, Yang G, Zhu K, Yin Z, Li Z, et al. Flexible mechanical metamaterials enabled electronic skin for real-time detection of unstable grasping in robotic manipulation. Adv Funct Mater. 2022;32(23):2109109.

[18] Fang H, Wang L, Fu Z, Xu L, Guo W, Huang J, Wang ZL, Wu H. Anatomically designed triboelectric wristbands with adaptive accelerated learning for human–machine interfaces. Adv Sci. 2023;10(6):2205960. 10.1002/advs.202205960 PMC9951357 36683215

[19] Abu-Halimah J, Majmudar A, Tian BZ. Chemical approaches to emerging advancements in deformable bioelectronics: Synthesis, device concepts, performance, and applications. CCS Chem. 2024;6(1):11–26.

[20] Chou N, Yoo S, Kim S. A largely deformable surface type neural electrode array based on PDMS. IEEE Trans Neural Syst Rehabil Eng. 2013;21(4):544–553. 22907973 10.1109/TNSRE.2012.2210560

[21] Wang C, Li X, Hu H, Zhang L, Huang Z, Lin M, Zhang Z, Yin Z, Huang B, Gong H, et al. Monitoring of the central blood pressure waveform via a conformal ultrasonic device. Nat Biomed Engr. 2018;2(9):687–695. 10.1038/s41551-018-0287-x PMC6428206 30906648

[22] Wang F, Wu D, Jin P, Zhang Y, Yang Y, Ma Y, Yang A, Fu J, Feng X. A flexible skin-mounted wireless acoustic device for bowel sounds monitoring and evaluation. Sci China Inf Sci. 2019;62(10).

[23] Dickey MD, Chiechi RC, Larsen RJ, Weiss EA, Weitz DA, Whitesides GM. Eutectic gallium-indium (EGaIn): A liquid metal alloy for the formation of stable structures in microchannels at room temperature. Adv Funct Mater. 2008;18(7):1097–1104.

[24] Xu Y, Rothe R, Voigt D, Hauser S, Cui M, Miyagawa T, Patino Gaillez M, Kurth T, Bornhäuser M, Pietzsch J, et al. Convergent synthesis of diversified reversible network leads to liquid metal-containing conductive hydrogel adhesives. Nat Commun. 2021;12(1). 10.1038/s41467-021-22675-2 PMC8065207 33893308

[25] Baharfar M, Kalantar-Zadeh K. Emerging role of liquid metals in sensing. ACS Sensors. 2022;7(2):386–408. 35119830 10.1021/acssensors.1c02606

[26] Chen F, Zhuang Q, Ding Y, Zhang C, Song X, Chen Z, Zhang Y, Mei Q, Zhao X, Huang Q, et al. Wet-adaptive electronic skin. Adv Mater. 2023;35(49):2305630. 10.1002/adma.202305630 37566544

[27] Park D-W, Schendel AA, Mikael S, Brodnick SK, Richner TJ, Ness JP, Hayat MR, Atry F, Frye ST, Pashaie R, et al. Graphene-based carbon-layered electrode array technology for neural imaging and optogenetic applications. Nat Commun. 2014;5(1):5258. 25327513 10.1038/ncomms6258 PMC4218963

[28] Jian M, Wang C, Wang Q, Wang H, Xia K, Yin Z, Zhang M, Liang X, Zhang Y. Advanced carbon materials for flexible and wearable sensors. Sci China Mater. 2017;60(11):1026–1062.

[29] Deng J, Yuk H, Wu J, Varela CE, Chen X, Roche ET, Guo CF, Zhao X. Electrical bioadhesive interface for bioelectronics. Nat Mater. 2021;20(2):229–236. 32989277 10.1038/s41563-020-00814-2

[30] Saadatnia Z, Ghaffarimosanenzadeh S, Marquez Chin M, Naguib HE, Popovic MR. Flexible, air dryable, and fiber modified aerogel-based wet electrode for electrophysiological monitoring. IEEE Trans Biomed Eng. 2021;68(6):1820–1827. 32897858 10.1109/TBME.2020.3022615

[31] Jiang S, Guo W, Liu S, Huang X, Li Y, Li Z, Wu H, Yin Z. Grab and heat: Highly responsive and shape adaptive soft robotic heaters for effective heating of objects of three-dimensional curvilinear surfaces. ACS Appl Mater Interfaces. 2019;11(50):47476–47484. 31765119 10.1021/acsami.9b19889

[32] Tringides CM, Boulingre M, Mooney DJ. Correction to: Metal-based porous hydrogels for highly conductive biomaterial scaffolds. Oxf Open Mater Sci. 2023;3(1): Article itad002. 38249777 10.1093/oxfmat/itad002 PMC10798674

[33] Wang X, Sun X, Gan D, Soubrier M, Chiang H-Y, Yan L, Li Y, Li J, Yu S, Xia Y, et al. Bioadhesive and conductive hydrogel-integrated brain-machine interfaces for conformal and immune-evasive contact with brain tissue. Matter. 2022;5(4):1204–1223.

[34] Jiang Y, Trotsyuk AA, Niu S, Henn D, Chen K, Shih C-C, Larson MR, Mermin-Bunnell AM, Mittal S, Lai J-C, et al. Wireless, closed-loop, smart bandage with integrated sensors and stimulators for advanced wound care and accelerated healing. Nat Biotechnol. 2023;41(5):652–662. 36424488 10.1038/s41587-022-01528-3

[35] Hui Y, Yao Y, Qian Q, Luo J, Chen H, Qiao Z, Yu Y, Tao L, Zhou N. Three-dimensional printing of soft hydrogel electronics. Nat Electron. 2022;5(12):893–903.

[36] Bo R, Xu S, Yang Y, Zhang Y. Mechanically-guided 3D assembly for architected flexible electronics. Chem Rev. 2023;123(18):11137–11189. 37676059 10.1021/acs.chemrev.3c00335 PMC10540141

[37] Jurinovs M, Barkane A, Platnieks O, Grase L, Gaidukovs S. Three dimensionally printed biobased electrodes: Ionic liquid and single-walled carbon nanotube hybrids in a vegetable oil matrix for soft robotics. ACS Appl Polym Mater. 2023;5(9):7120–7131.

[38] Hiendlmeier L, Zurita F, Vogel J, Del Duca F, Al Boustani G, Peng H, Kopic I, Nikić M. 4D-printed soft and stretchable self-folding cuff electrodes for small-nerve interfacing. Adv Mater. 2023;35(12):2210206. 10.1002/adma.202210206 36594106

[39] Ji Y, Liao Y, Li H, Cai Y, Fan D, Liu Q, Huang S, Zhu R, Wang S, Wang H, et al. Flexible metal electrodes by femtosecond laser-activated deposition for human–Machine interfaces. ACS Appl Mater Interfaces. 2022;14(9):11971–11980. 35212517 10.1021/acsami.2c00419

[40] Yang K, Freeman C, Torah R, Beeby S, Tudor J. Screen printed fabric electrode array for wearable functional electrical stimulation. Sens Actuators A Phys. 2014;213:108–115.

[41] Merhi Y, Betancur PF, Ripolles TS, Suetta C, Brage-Andersen MR, Hansen SK, Frydenlund A, Nygaard JV, Mikkelsen PH, Boix PP, et al. Printed dry electrode for neuromuscular electrical stimulation (NMES) for e-textile. Nanoscale. 2023;15(11):5337–5344. 36815314 10.1039/d2nr06008f

[42] Xue H, Wang D, Jin M, Gao H, Wang X, Xia L, Li DA, Sun K, Wang H, Dong X, et al. Hydrogel electrodes with conductive and substrate-adhesive layers for noninvasive long-term EEG acquisition. Microsyst Nanoeng. 2023;9(1): Article 79. 37313471 10.1038/s41378-023-00524-0 PMC10258200

[43] Yang G, Hu Y, Guo W, Lei W, Liu W, Guo G, Geng C, Liu Y, Wu H. Tunable hydrogel electronics for diagnosis of peripheral neuropathy. Adv Mater. 2024;36(18): Article e2308831. 37906182 10.1002/adma.202308831

[44] Niu X, Gao X, Liu Y, Liu H. Surface bioelectric dry electrodes: A review. Measurement. 2021;183: Article 109774.

[45] Nucci NV, Vanderkooi JM. Effects of salts of the Hofmeister series on the hydrogen bond network of water. J Mol Liq. 2008;143(2-3):160–170. 19847287 10.1016/j.molliq.2008.07.010 PMC2748947

[46] Zhou C, Zhao X, Xiong Y, Tang Y, Ma X, Tao Q, Sun C, Xu W. A review of etching methods of MXene and applications of MXene conductive hydrogels. Eur Polym J. 2022;167: Article 111063.

[47] Yang G, Zhu K, Guo W, Wu D, Quan X, Huang X, Liu S, Li Y, Fang H, Qiu Y, et al. Adhesive and hydrophobic bilayer hydrogel enabled on-skin biosensors for high-fidelity classification of human emotion. Adv Funct Mater. 2022;32(29):2200457.

[48] He Q, Huang Y, Wang S. Hofmeister effect-assisted one step fabrication of ductile and strong gelatin hydrogels. Adv Funct Mater. 2018;28(5):1705069.

[49] Wu S, Hua M, Alsaid Y, Du Y, Ma Y, Zhao Y, Lo CY, Wang C, Wu D, Yao B, et al. Poly(vinyl alcohol) hydrogels with broad-range tunable mechanical properties via the Hofmeister effect. Adv Mater. 2021;33(11):2007829. 10.1002/adma.202007829 33554414

[50] Hua M, Wu S, Ma Y, Zhao Y, Chen Z, Frenkel I, Strzalka J, Zhou H, Zhu X, He X. Strong tough hydrogels via the synergy of freeze-casting and salting out. Nature. 2021;590(7847):594–599. 33627812 10.1038/s41586-021-03212-z

[51] Zhang Y-Z, El-Demellawi JK, Jiang Q, Ge G, Liang H, Lee K, Dong X, Alshareef HN. MXene hydrogels: Fundamentals and applications. Chem Soc Rev. 2020;49(20):7229–7251. 32936169 10.1039/d0cs00022a

[52] Tang H, Li Y, Liao S, Liu H, Qiao Y, Zhou J. Multifunctional conductive hydrogel interface for bioelectronic recording and stimulation. Adv Healthc Mater. 2024; Article 2400562. 10.1002/adhm.202400562 38773929

[53] Yao B, Wu S, Wang R, Yan Y, Cardenas A, Wu D, Alsaid Y, Wu W, Zhu X, He X. Hydrogel ionotronics with ultra-low impedance and high signal fidelity across broad frequency and temperature ranges. Adv Funct Mater. 2022;32(10):2109506.

