# Supplementary material for: Hoffmeister Effect Optimized Hydrogel Electrodes with Enhanced Electrical and Mechanical Properties for Nerve Conduction Studies
Source: Research (Wash D C). 2024 Aug 14;7:0453. doi: 10.34133/research.0453 (PMC11322598; doi:10.34133/research.0453)
Supplement: Supplementary 1 — Figs. S1 to S10 Tables S1 to S4 Note S1 [file research.0453.f1.zip › Supplementary Materials.docx]

Supplementary Materials


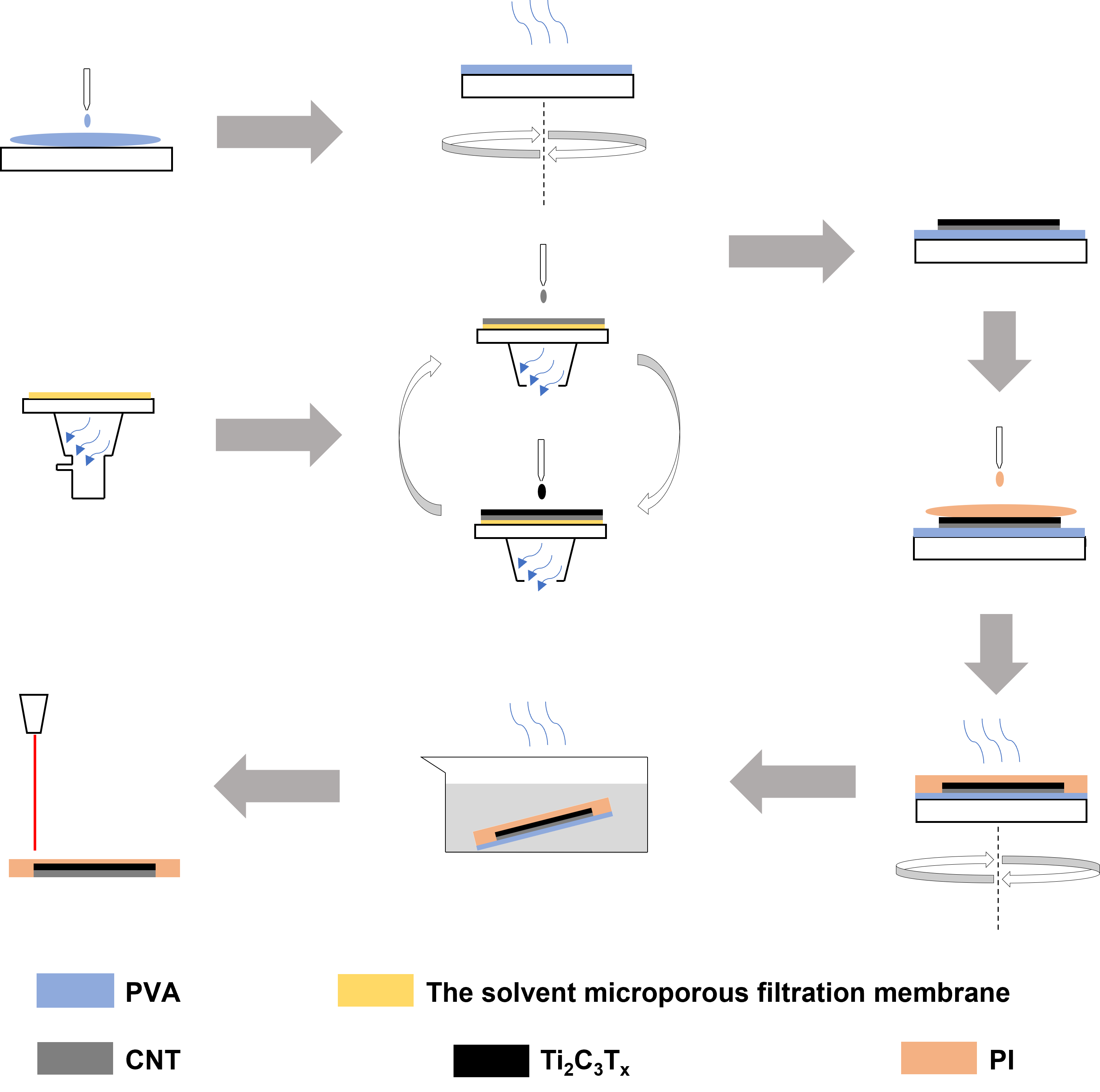


Fig. S1. Schematic illustration of the fabrication processes of the CCL.


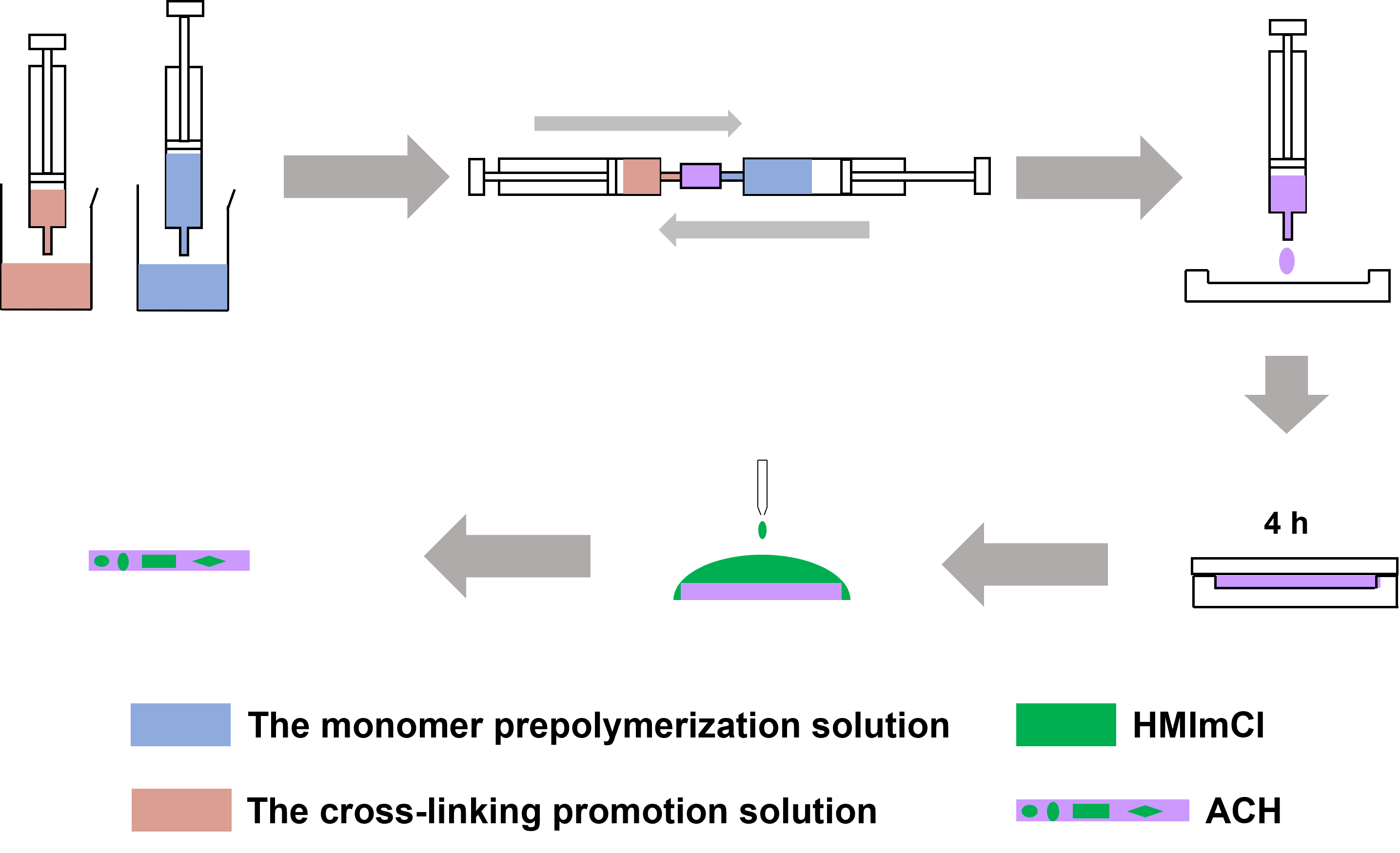


Fig. S2. Schematic illustration of the fabrication processes of the ACH.


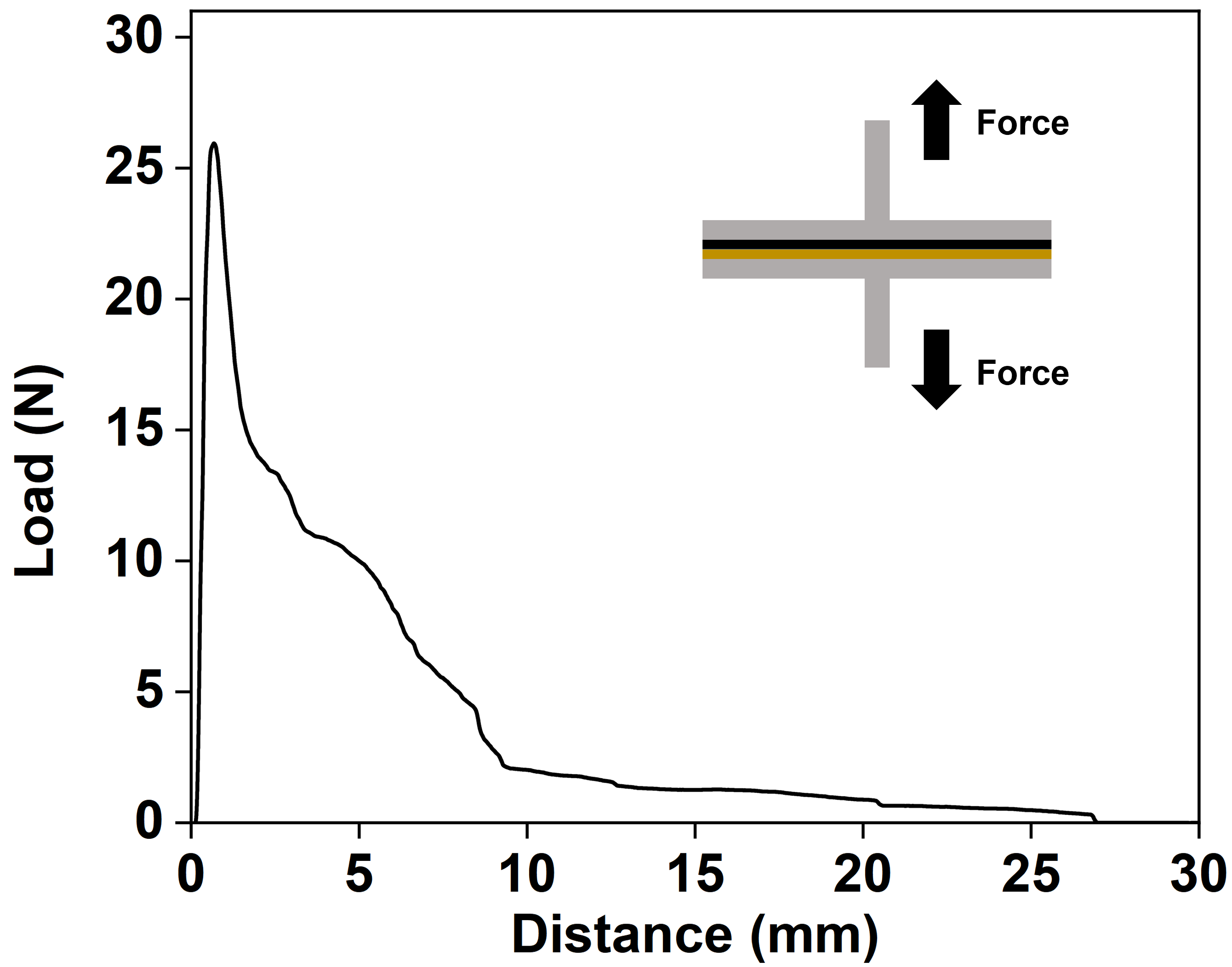


Fig. S3. The load-distance curves between the ACH and the CCL. Inset, schematic of the experimental condition.


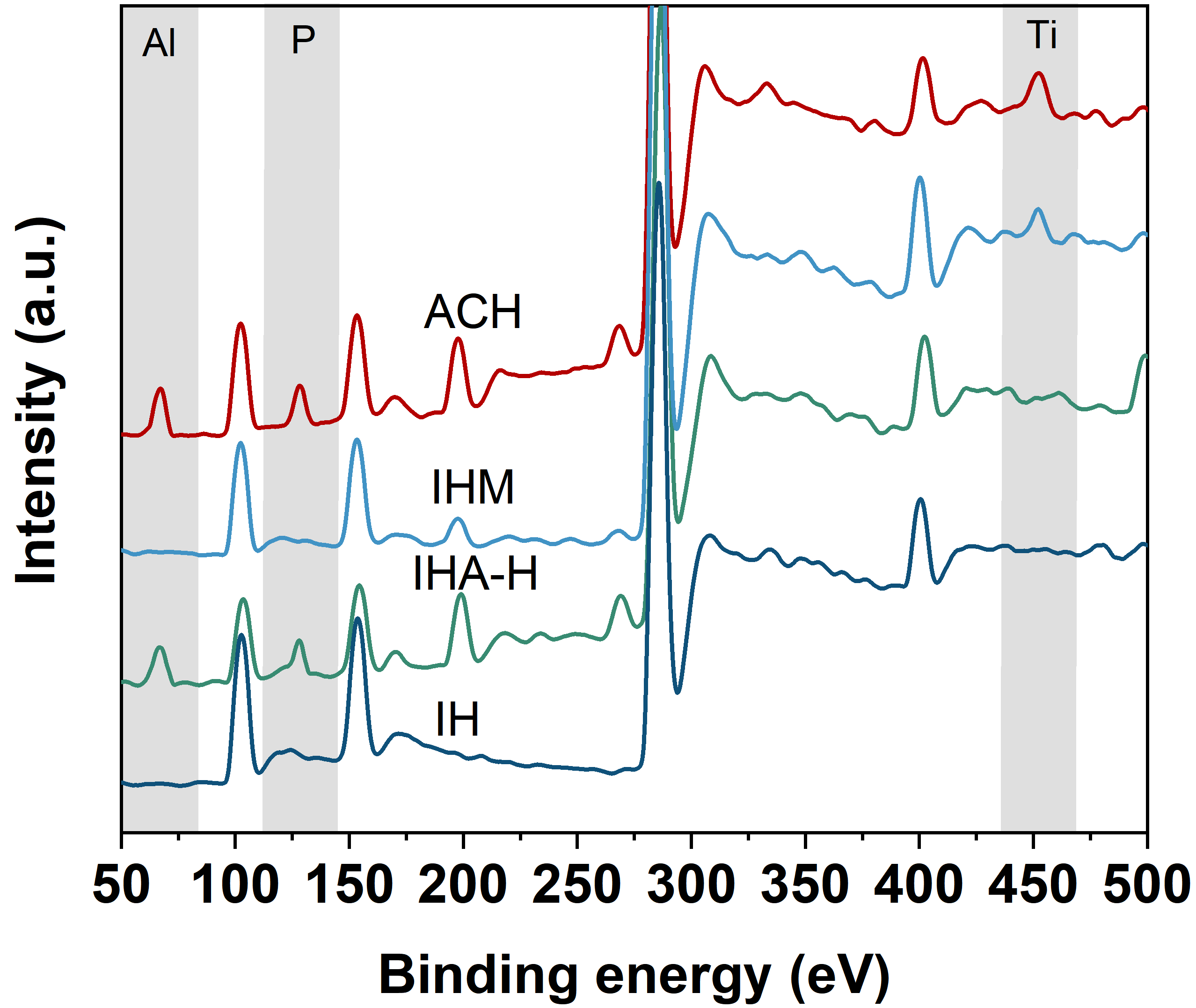


Fig. S4. XPS total scan spectra of IH, IHA-H, IHM and ACH.


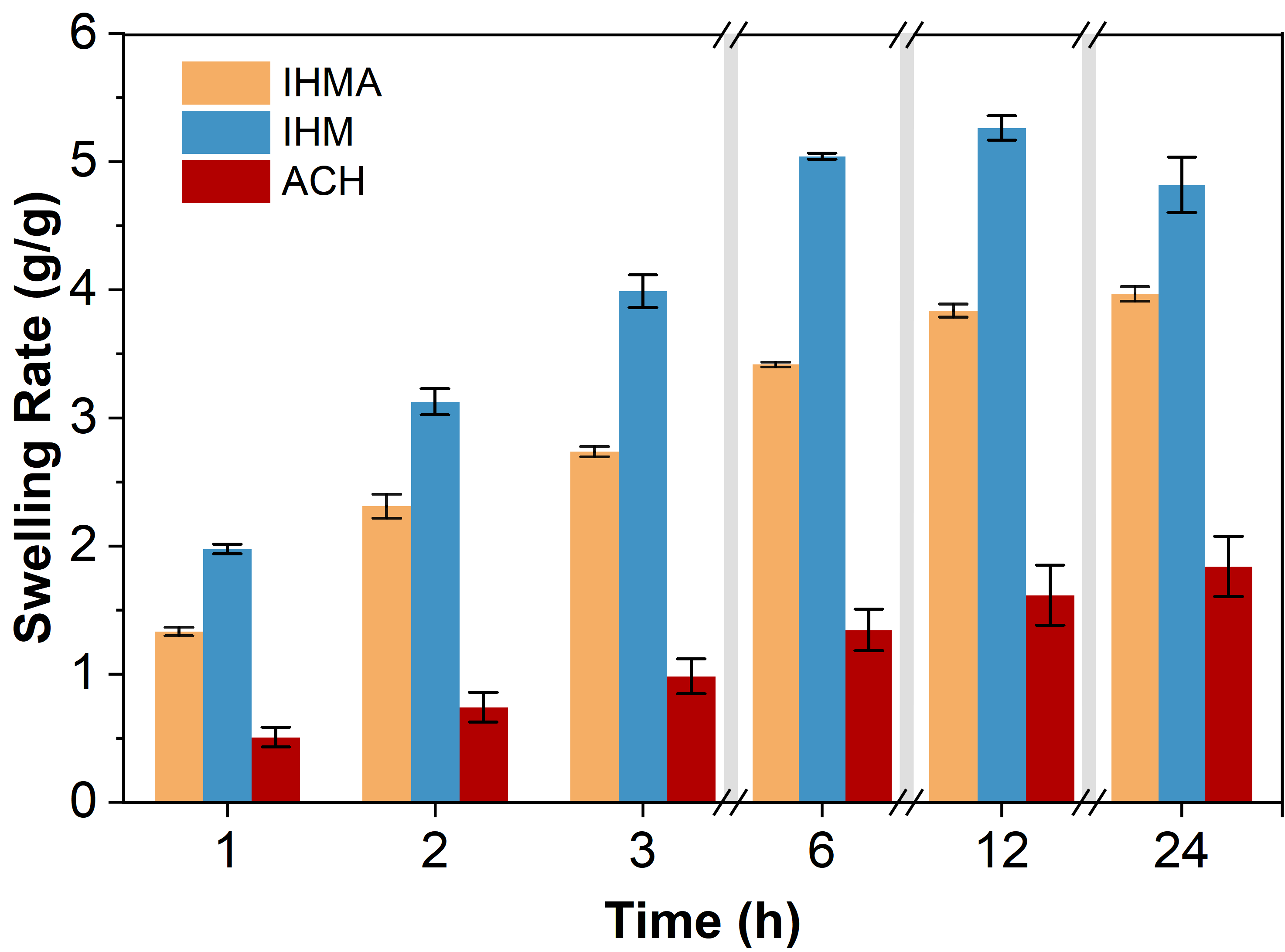


Fig. S5. Swelling rate-time variation of IHM, IHMA and ACH, demonstrating that PO_4_^3-^ can reduce the swelling rate of the hydrogel.


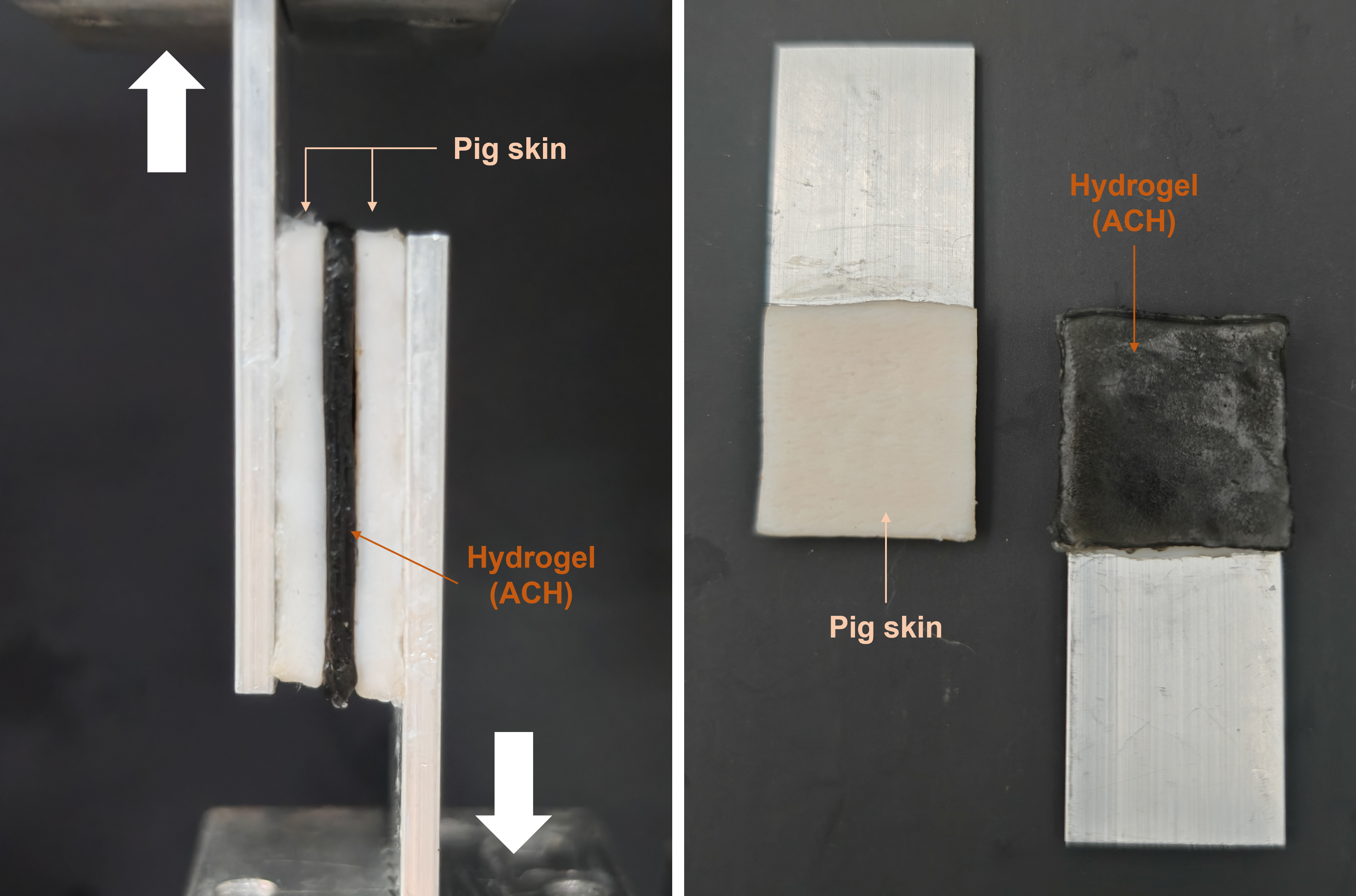


Fig. S6. Hydrogel adhesion tests with freshly dehairing pig skin as substrate.


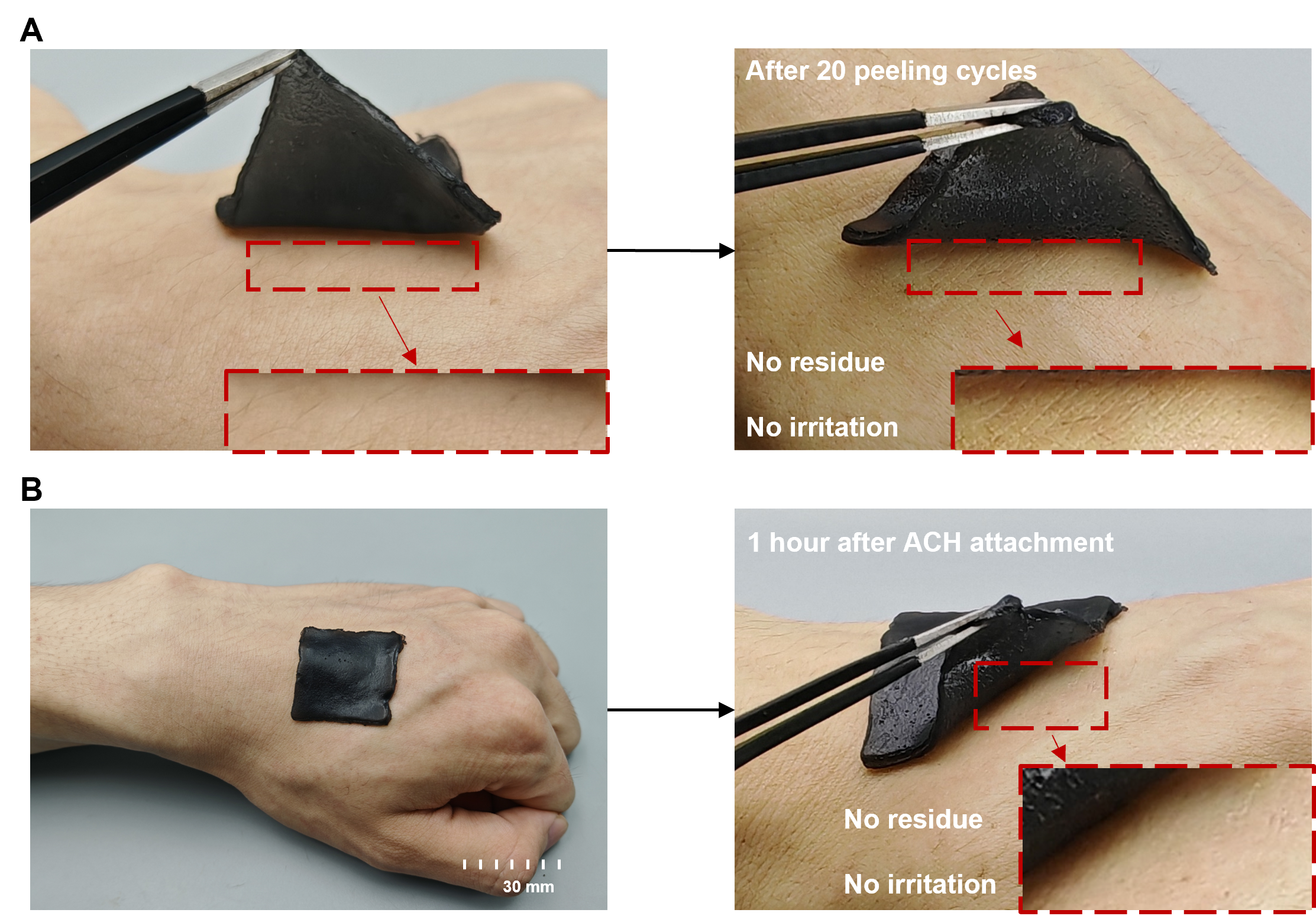


Fig. S7. The condition of human skin after 20 peeling cycles or 1 hour of ACH attachment, and there is no residue or irritation.


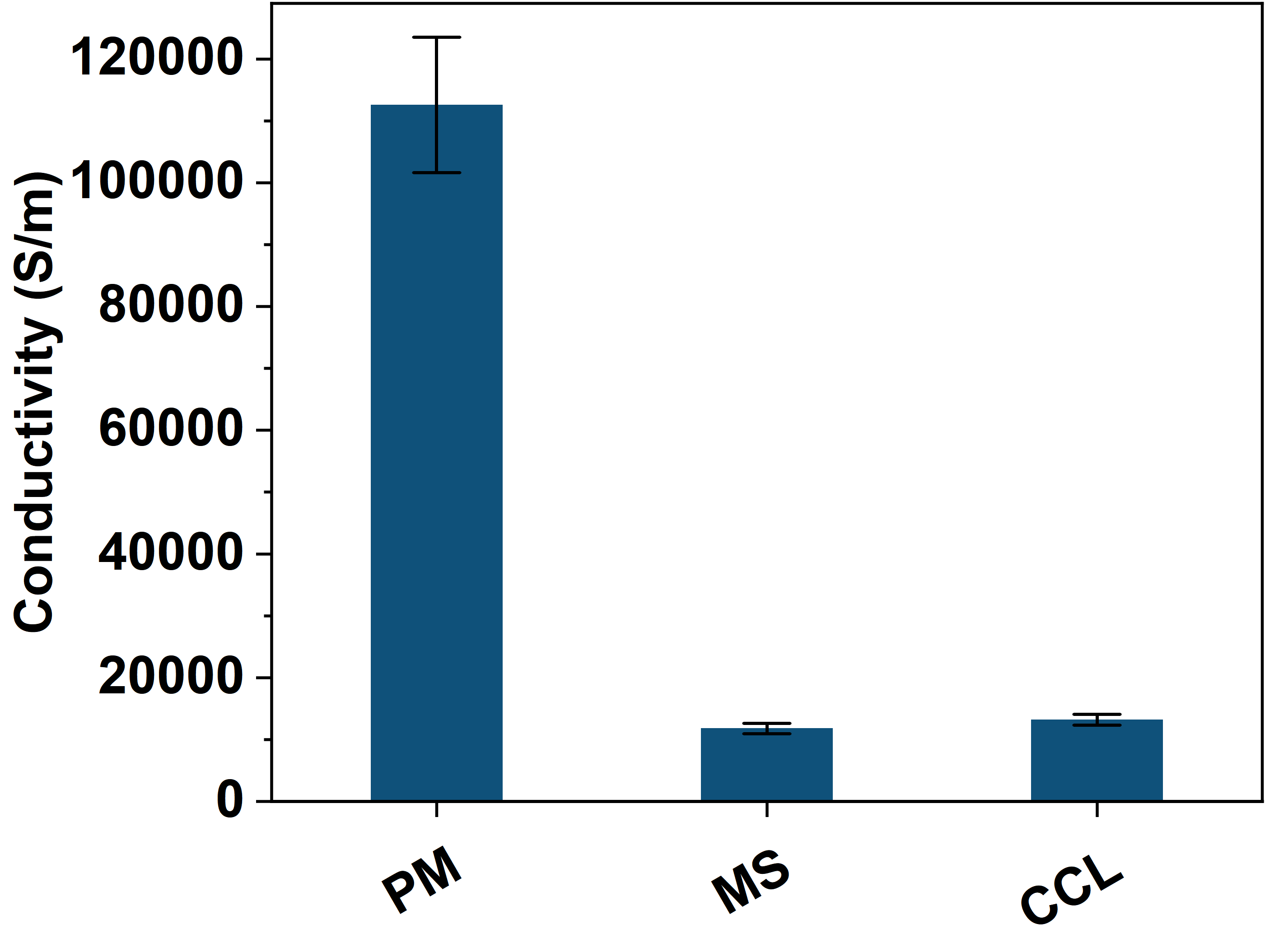


Fig. S8. Comparison of conductivity of PM, MS and CCL.


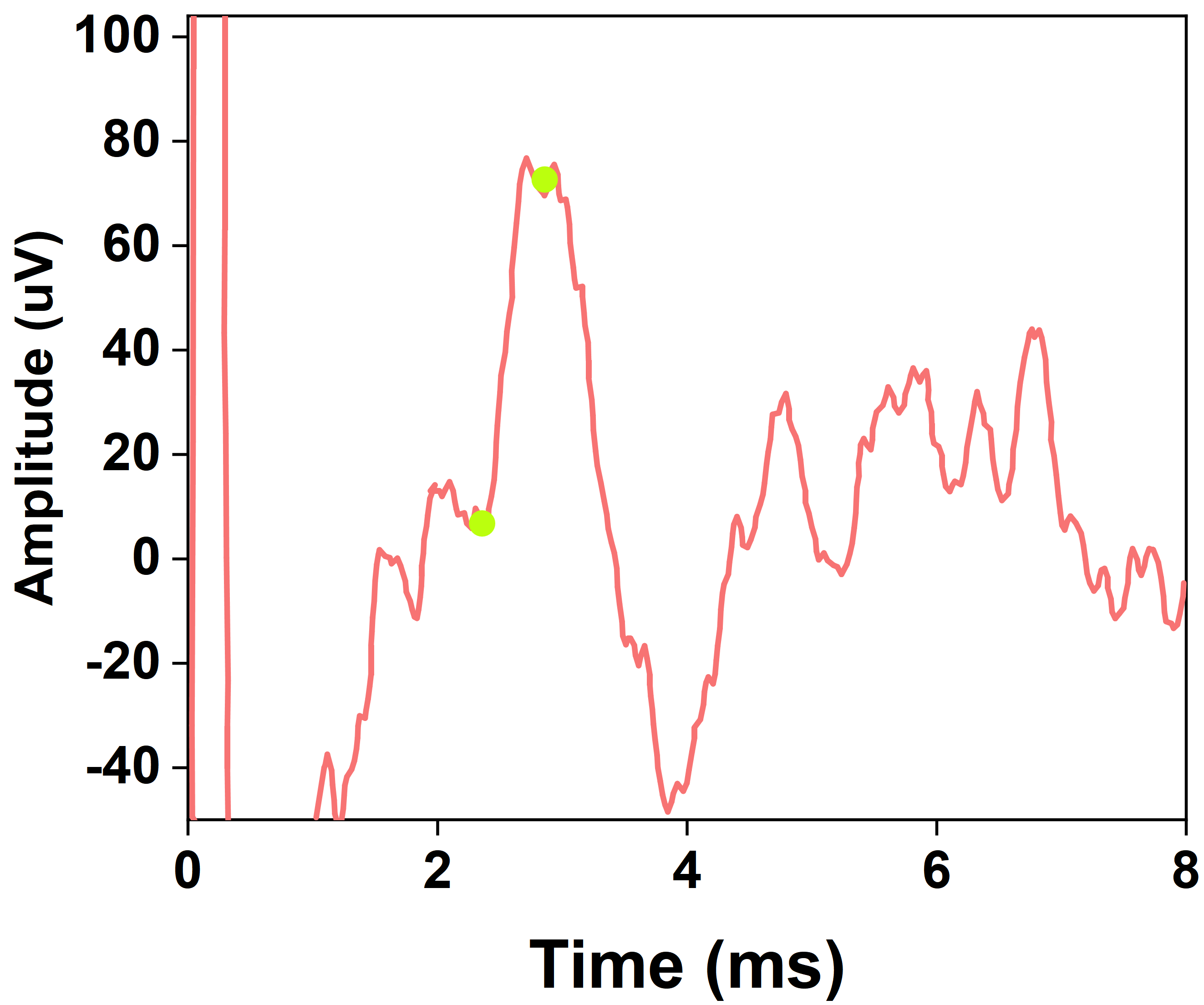


Fig. S9. Waveform images of sensory branches of the median nerve acquired by commercial Ag/AgCl gel electrodes.


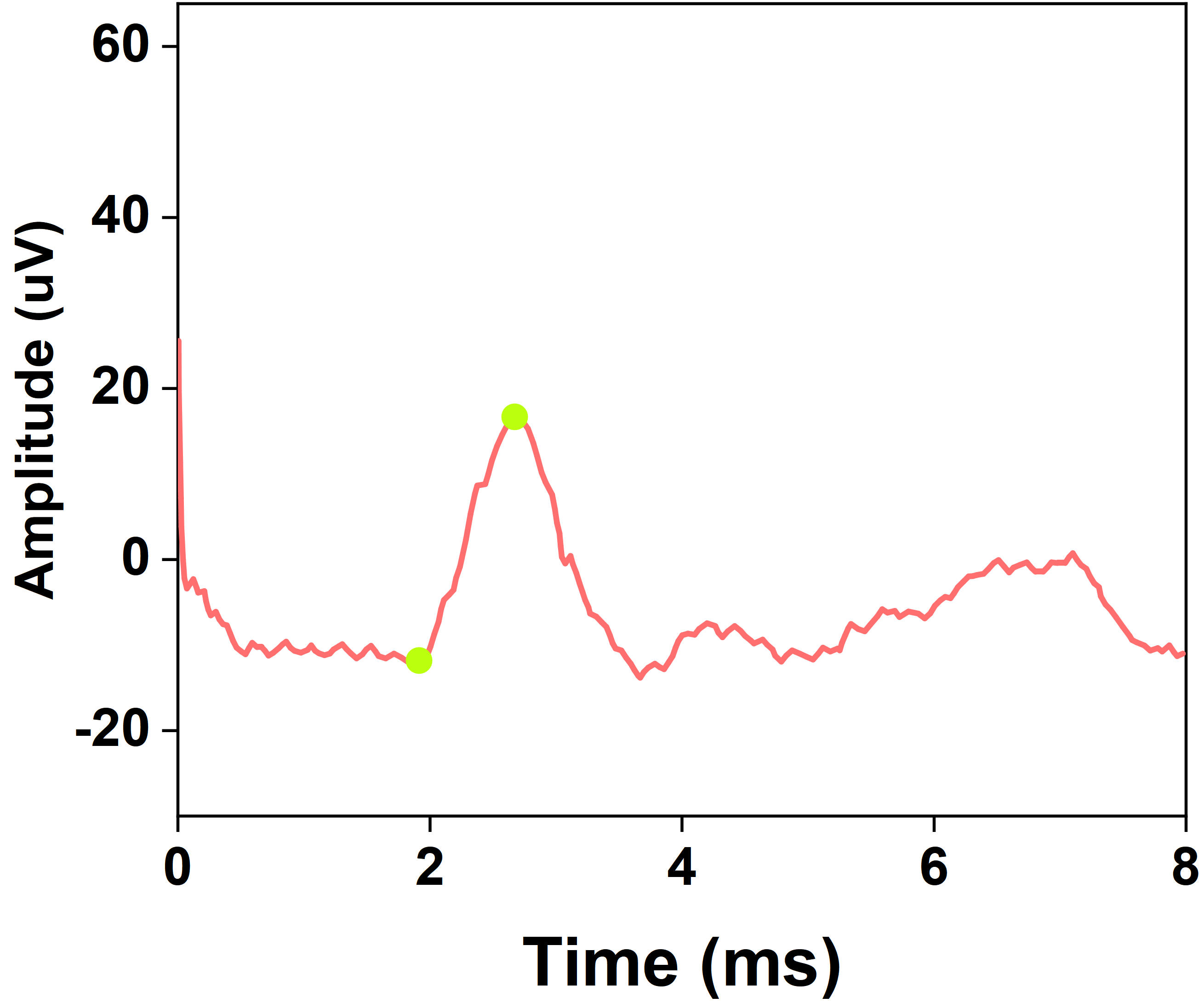


Fig. S10. Waveform images of sensory branches of the ulnar nerve acquired by commercial Ag/AgCl gel electrodes.

Table S1. Distal amplitude, proximal amplitude, conduction velocity, and distal motor latency of the motor branch of the median nerve in volunteers measured by electromyography.

| Distal amplitude (mV) | Proximal amplitude (mV) | Conduction velocity (m/s) | Distal motor latency (ms) |
| --- | --- | --- | --- |
| 9.6 | 10 | 58.8 | 3.2 |

Table S2. Amplitude, sensory latency, and conduction velocity in volunteers of the sensory branch of the median nerve in volunteers measured by electromyography.

| Amplitude (uV) | Sensory latency (ms) | Conduction velocity (m/s) |
| --- | --- | --- |
| 73 | 3.1 | 60.4 |

Table S3. Distal amplitude, proximal amplitude, conduction velocity, and distal motor latency of the motor branch of the ulnar nerve in volunteers measured by electromyography.

| Distal amplitude (mV) | Proximal amplitude (mV) | Conduction velocity (m/s) | Distal motor latency (ms) |
| --- | --- | --- | --- |
| 8.1 | 8.3 | 54.1 | 2.4 |

Table S4. Amplitude, sensory latency, and conduction velocity in volunteers of the sensory branch of the ulnar nerve in volunteers measured by electromyography.

| Amplitude (uV) | Sensory latency (ms) | Conduction velocity (m/s) |
| --- | --- | --- |
| 36 | 1.82 | 61.2 |

Note S1.

Observing from left to right in the FTIR-ATR, the wavelengths change from long to short, and it can be found that there are absorption peaks at 3332 cm^-1^ and 3167 cm^-1^ for both IH and IHA-H, which are the result of the symmetric stretching vibration of the amino group (N-H) here. Among them, the IHA-H group has larger peaks than the IH, which proves that HMImCl can provide more amino groups. After the introduction of MXene, the peak of the vibrational band at 3332 cm^-1^ of the PMSI group was shifted to 3350 cm^-1^, and the IHM was even more directly devoid of absorption peaks in the range of 3000~3500 cm^-1^, which indicated that the incorporation of Ti_3_C_2_T_x_ attenuates the N-H stretching vibration. The reason is presumed to be the large number of hydroxyl groups (-OH) on the surface of Ti_3_C_2_T_x_ forming hydrogen bonds with N-H. This result also implies the strong interaction between Ti_3_C_2_T_x_ and the hydrogel network, which laterally confirms the enhancement of the mechanical properties of the gels by Ti_3_C_2_T_x_. Two other examples of the above speculation are the bending vibrational peak of N-H at 1600 cm^-1^ and the planar bending vibrational peak of N-H at 1165 cm^-1^, where the peaks are larger for both groups with the addition of IL; all other things being equal, the peaks are larger for both groups with the addition of Ti_3_C_2_T_x_, on the other hand, the peaks of the two groups with the addition of Ti_3_C_2_T_x_ are significantly weakened.

Continuing to the right, both the IHA-H and ACH groups have more pronounced absorption peaks at 2960 cm^-1^ and 2860 cm^-1^ produced by the asymmetric stretching vibration of methylene (-CH2-) than the IH and IHM. HMImCl has a lot of methylene groups, which is a side effect of the fact that the amount of ionic liquid entering the hydrogel system after solvent substitution is quite high. The large absorption peak at 1658 cm-1 in the IHA-H and ACH is the stretching vibration band of the carbonyl group (C=O), which is similarly missing in the IH and IHM because of the absence of HMImCl. 1450 cm^-1^ is the stretching vibration band of -CH2-. 1415 cm-^-1^, 1321 cm^-1^ and 1280 cm^-1^ are the stretching vibration bands of the nitrile group (C-N) stretching vibration, all groups have this peak, which has two sources, the nitrile group carried by AAm and HMImCl, and the nitrile group formed by the reaction between the amino group in AAm and the carboxyl group (-COOH) in SA. Similarly, the hydroxyl group on the surface of Ti_3_C_2_T_x_ consumes the amino group and reduces this effect, but the absorption peak does not disappear completely as in the range of 3000~3500 cm^-1^ due to the presence of the nitrile group inherent in AAm.

Each element (except H and He) has its own strongest and characteristic photoelectron line, which is the main basis for elemental qualitative analysis, among which the binding energy of elemental P is 128.53 eV, that of elemental Ti is 452.43 eV, and that of elemental Al is 67.38 eV, which are marked with grey rectangles in the figure, respectively. From the XPS, it can be found that both IH and IHM do not contain elemental Al and P, while IHA-H and ACH groups contain elemental Al and P, and their only source is the salt AlPO_4,_ in addition, the heights of the characteristic peaks of elemental Al and elemental P are similar, which is as expected because of their identical molar amounts in AlPO_4_ and the similar atomic weights of P and Al. The Ti element, on the other hand, appears only in the IHM and ACH groups, which is directly related to the presence or absence of Ti_3_C_2_T_x_. Organic matter accounts for the majority of the hydrogel, so the largest and highest peaks in all four groups are element C, with a binding energy of 285.03 eV.
