# Supplementary figures and images for: Hoffmeister Effect Optimized Hydrogel Electrodes with Enhanced Electrical and Mechanical Properties for Nerve Conduction Studies
Source: Research (Wash D C). 2024 Aug 14;7:0453. doi: 10.34133/research.0453 (PMC11322598; doi:10.34133/research.0453)

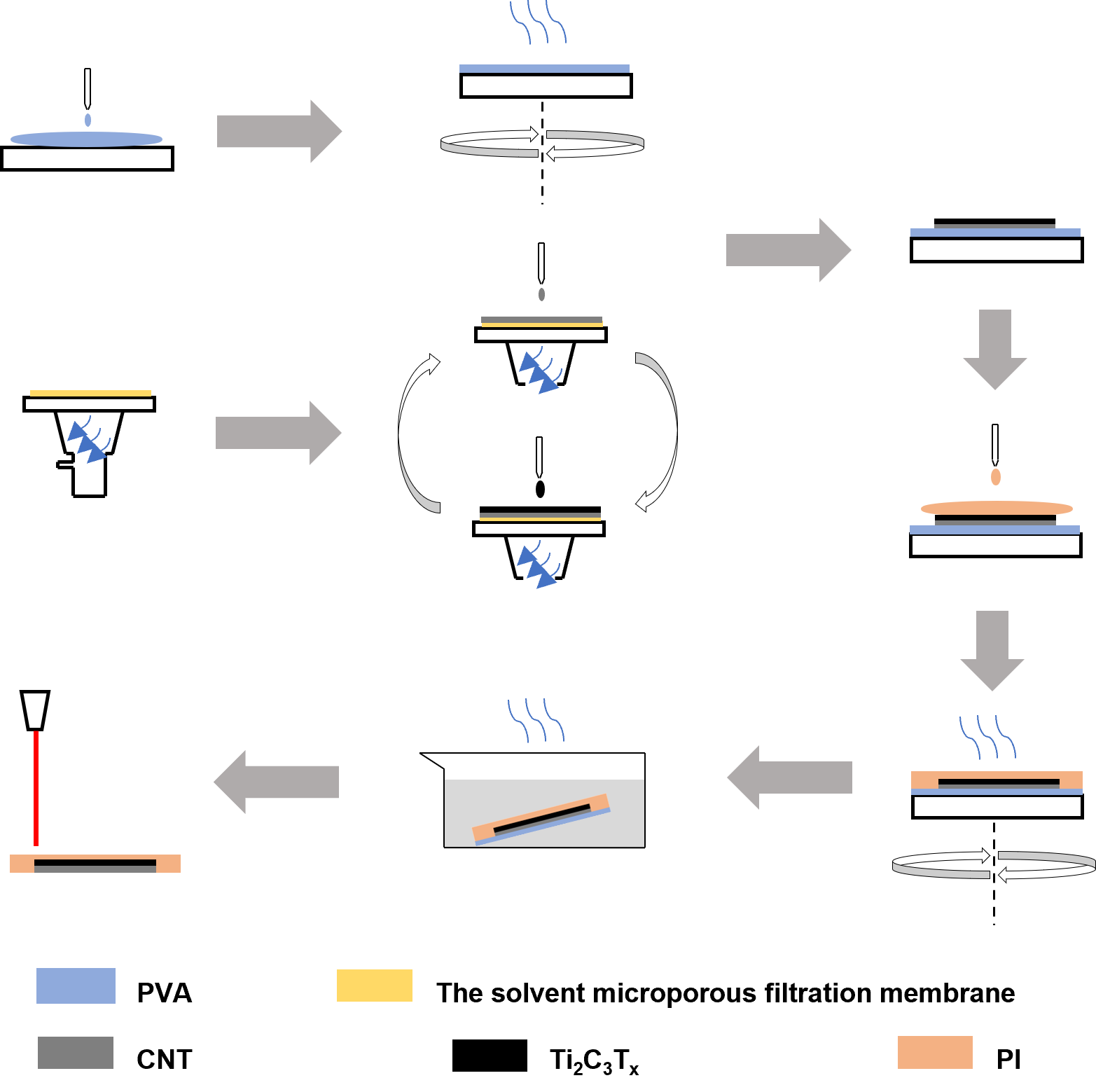

Supplement: Supplementary 1 — Figs. S1 to S10 Tables S1 to S4 Note S1 [file research.0453.f1.zip › Fig. S1.tif]

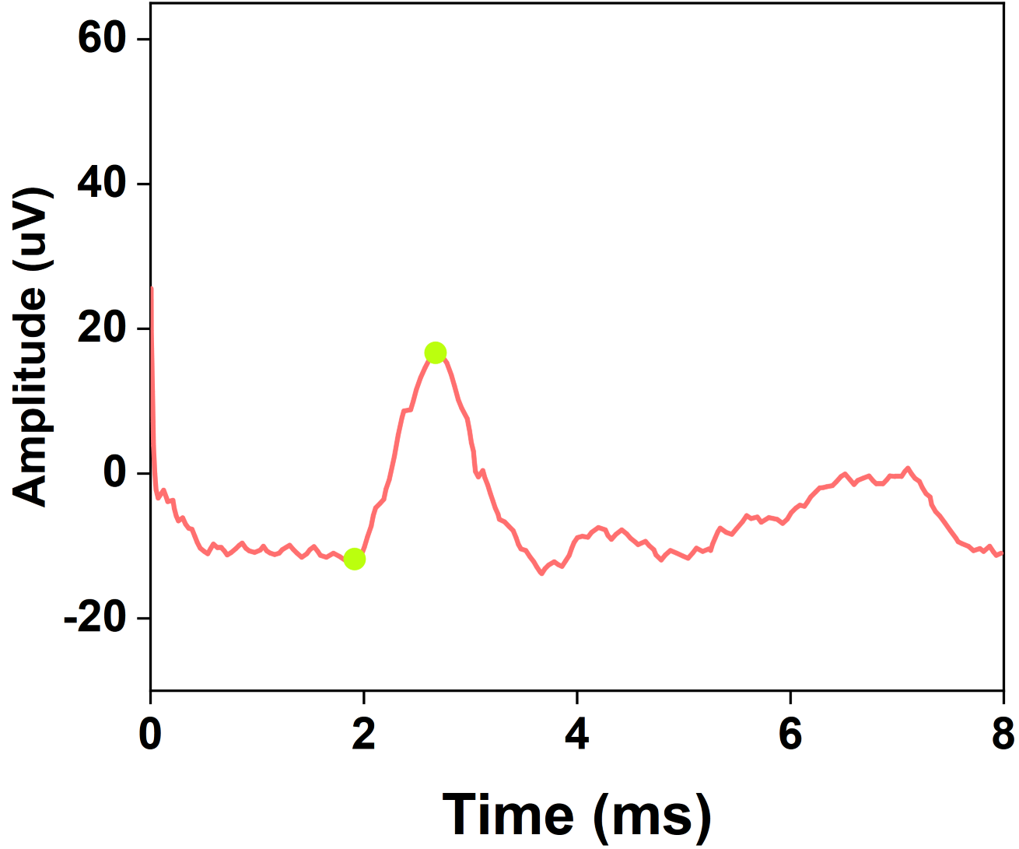

Supplement: Supplementary 1 — Figs. S1 to S10 Tables S1 to S4 Note S1 [file research.0453.f1.zip › Fig. S10.tif]

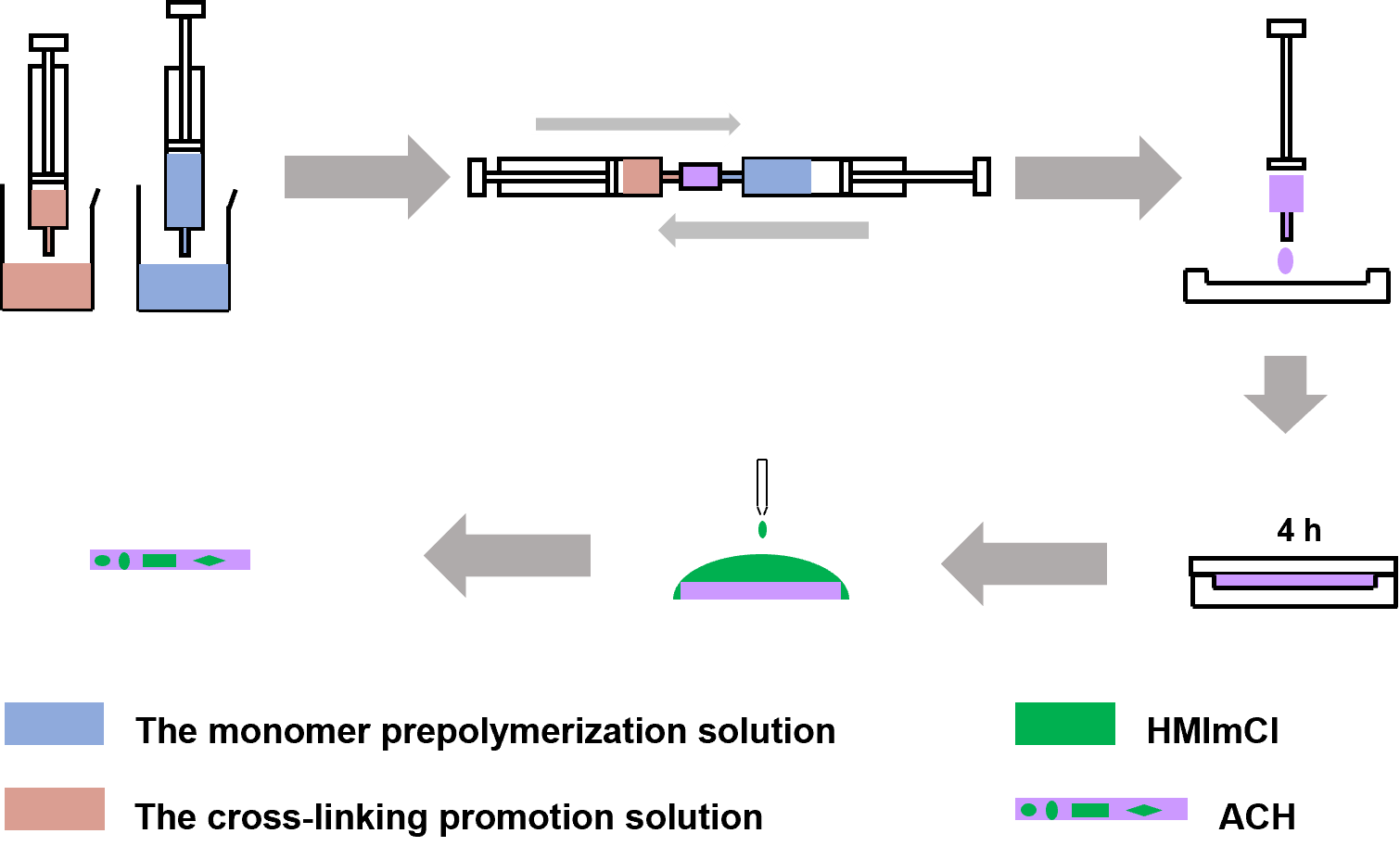

Supplement: Supplementary 1 — Figs. S1 to S10 Tables S1 to S4 Note S1 [file research.0453.f1.zip › Fig. S2.tif]

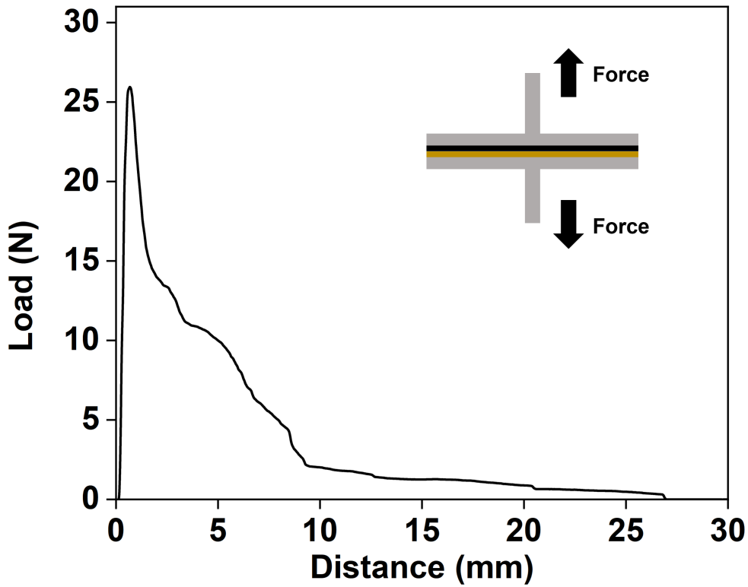

Supplement: Supplementary 1 — Figs. S1 to S10 Tables S1 to S4 Note S1 [file research.0453.f1.zip › Fig. S3.tif]

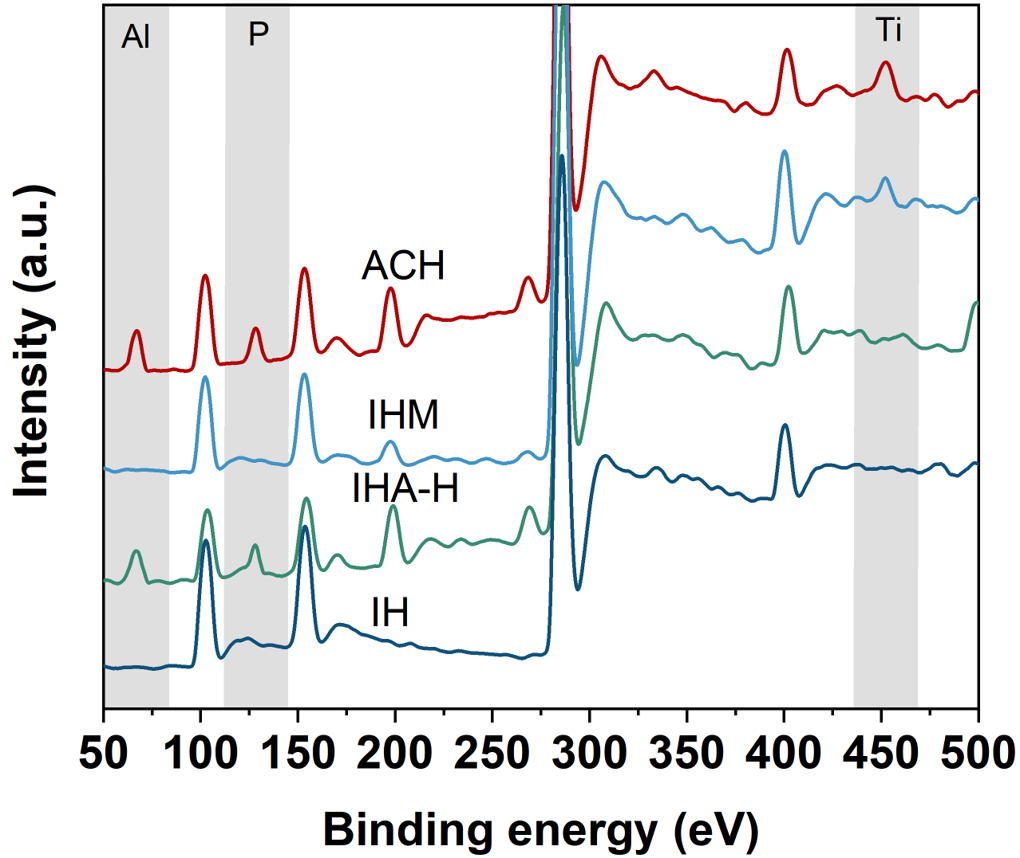

Supplement: Supplementary 1 — Figs. S1 to S10 Tables S1 to S4 Note S1 [file research.0453.f1.zip › Fig. S4.tif]

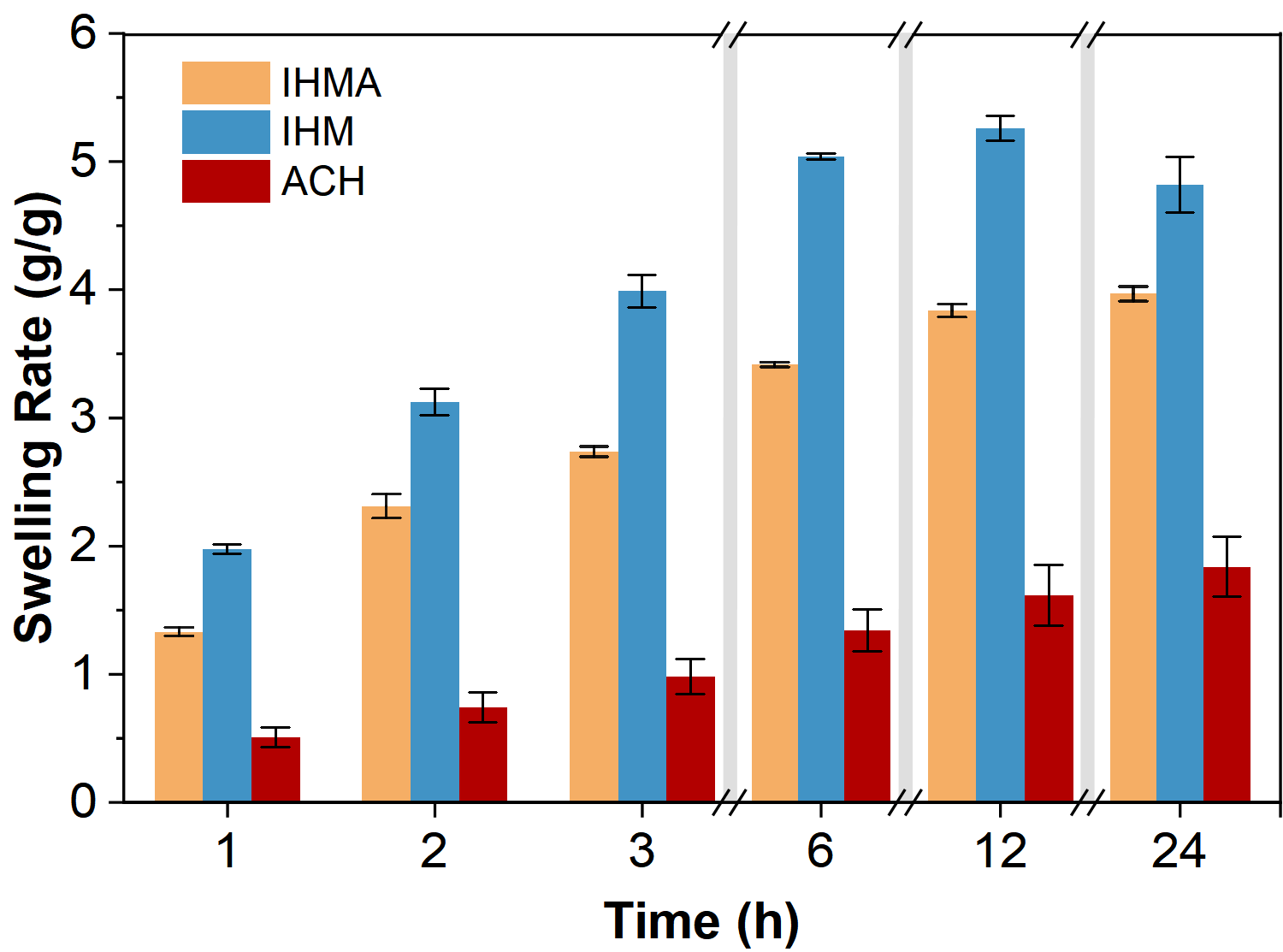

Supplement: Supplementary 1 — Figs. S1 to S10 Tables S1 to S4 Note S1 [file research.0453.f1.zip › Fig. S5.tif]

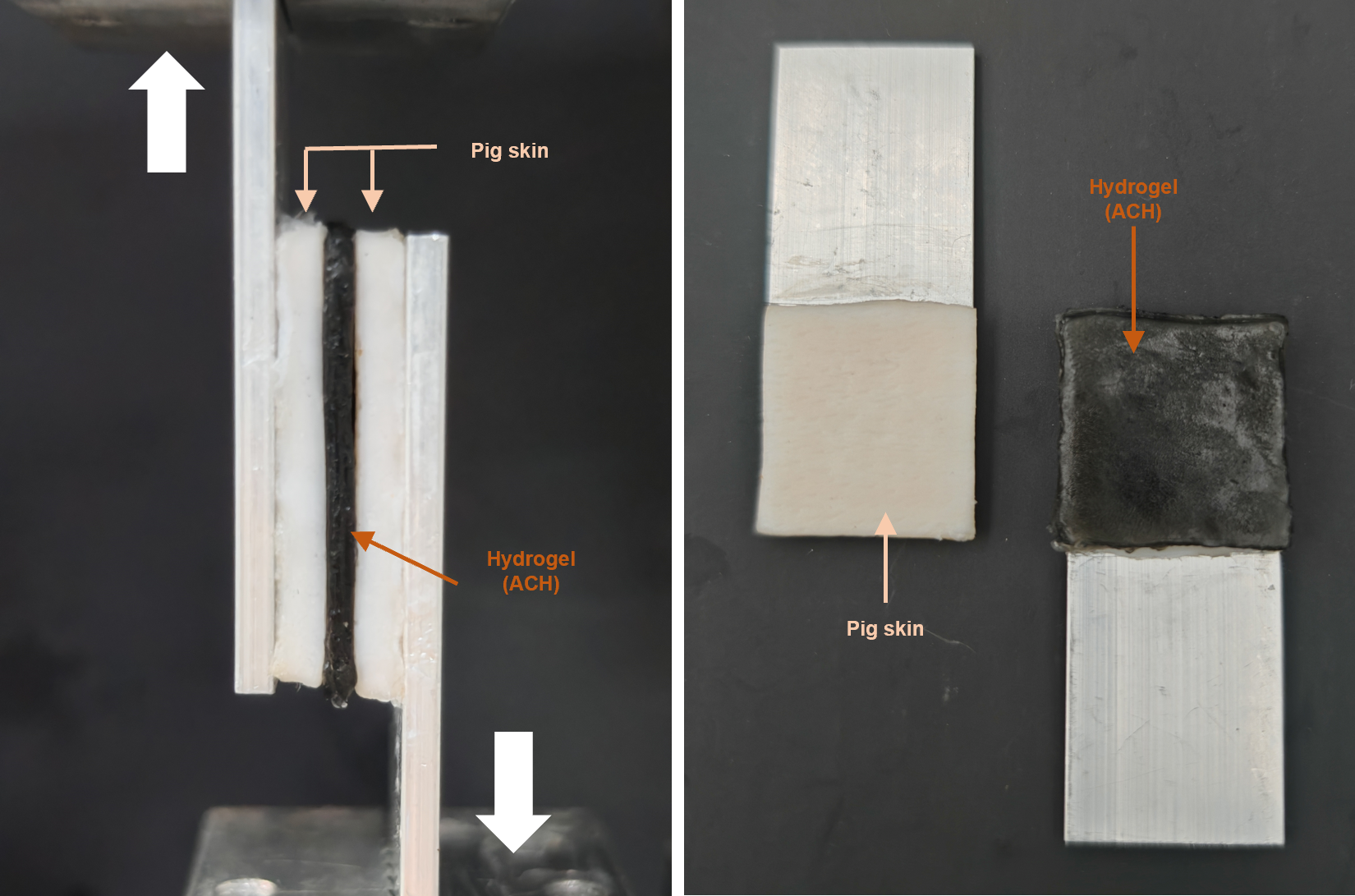

Supplement: Supplementary 1 — Figs. S1 to S10 Tables S1 to S4 Note S1 [file research.0453.f1.zip › Fig. S6.tif]

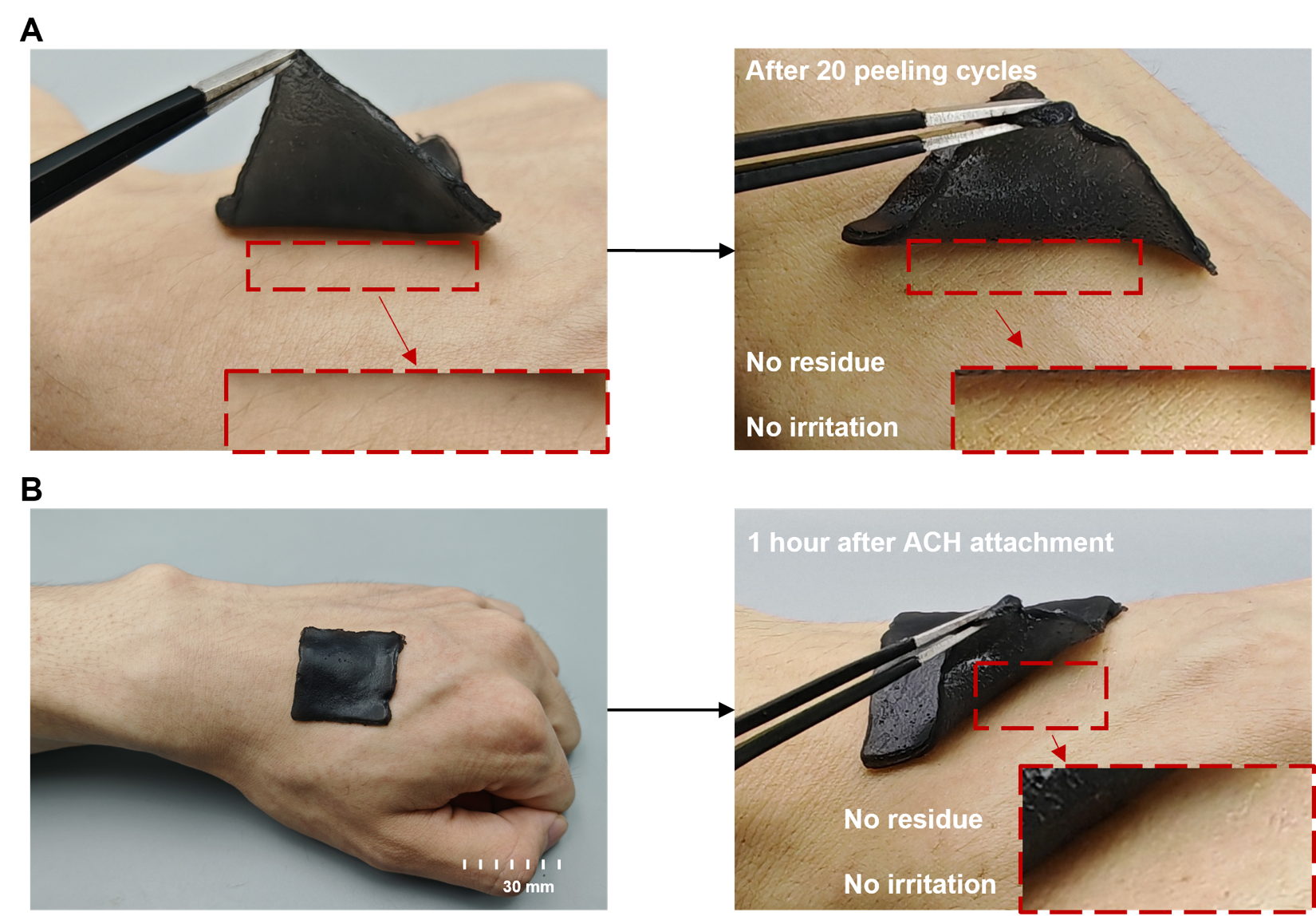

Supplement: Supplementary 1 — Figs. S1 to S10 Tables S1 to S4 Note S1 [file research.0453.f1.zip › FIg. S7.tif]

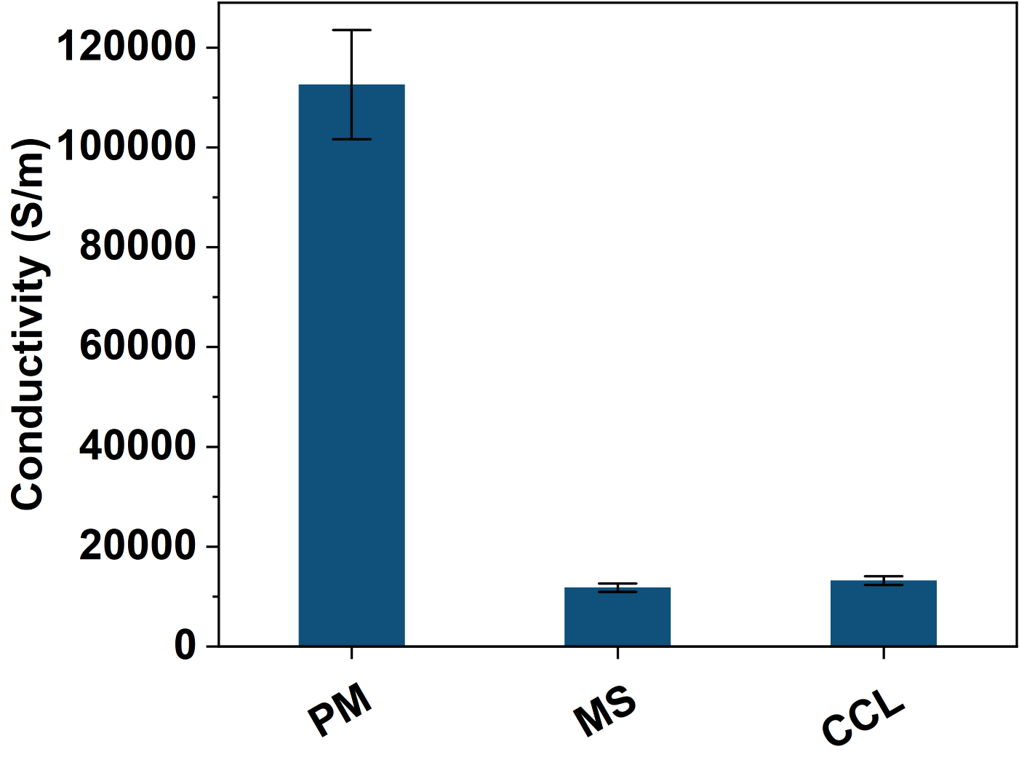

Supplement: Supplementary 1 — Figs. S1 to S10 Tables S1 to S4 Note S1 [file research.0453.f1.zip › Fig. S8.tif]

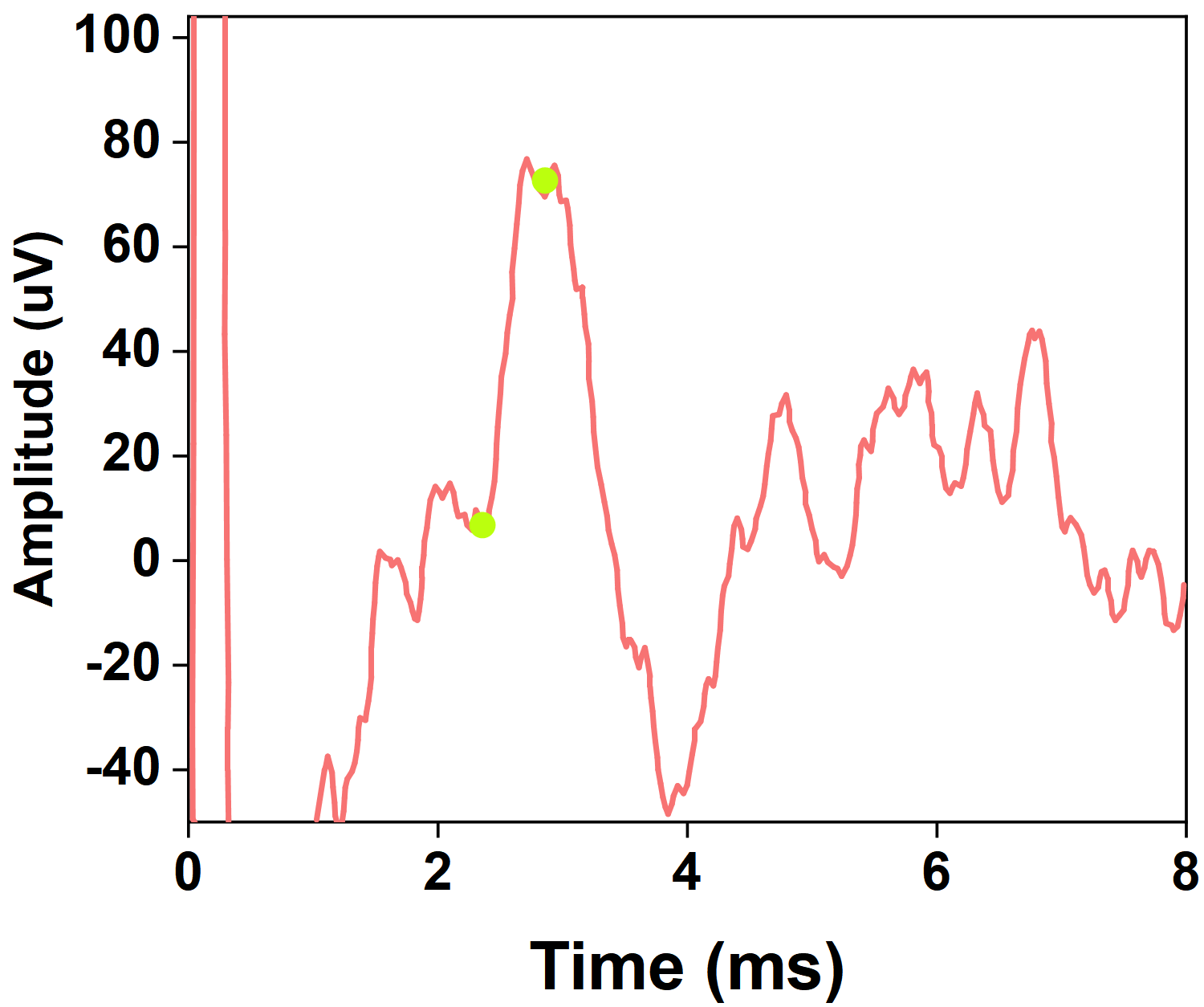

Supplement: Supplementary 1 — Figs. S1 to S10 Tables S1 to S4 Note S1 [file research.0453.f1.zip › Fig. S9.tif]
